# Quantum-enhanced trusted execution with dynamic policy optimization for zero-trust edge computing

**DOI:** 10.1016/j.isci.2026.117784

**Published:** 2026-11-02

**Authors:** Yi Liu, Yuanju Zhou

**Affiliations:** 1 School of Artificial Intelligence, Yibin University, Yibin 644100, China; 2 School of Artificial Intelligence and Electronic Engineering, Sichuan Technology and Business University, Chengdu 611745, China

**Keywords:** quantum-enhanced trusted execution environment, post-quantum cryptography, zero-trust edge computing, dynamic policy optimization, heterogeneous edge systems

## Abstract

Zero-trust edge computing faces escalating security challenges from quantum threats, heterogeneous resources, and dynamic operational conditions. This paper proposes Q-TEE-DSO, a collaborative architecture integrating quantum-enhanced trusted execution environments with post-quantum cryptography and reinforcement learning-based dynamic security optimization. By embedding quantum random number generation and lightweight quantum key distribution into hardware-enforced TEEs, the framework strengthens trust-root initialization and session-key establishment under quantum-aware threat models. Instruction-level optimization of lattice-based PQC algorithms, combined with enclave-aware memory isolation and side-channel mitigation, reduces cryptographic overhead on constrained edge devices. A reinforcement learning-driven DSO-Engine dynamically adapts encryption strength, access control policies, and privacy-preserving computation modes according to real-time system load and threat levels. Experimental evaluations on heterogeneous testbeds demonstrate a 37% reduction in cryptographic latency and 42% improvement in energy efficiency compared with conventional approaches, alongside 99.88% availability, robust heterogeneous compatibility, and stable long-term operation across diverse network topologies.

## Highlights

• Quantum-enhanced TEE integrates QRNG/QKD trust roots with post-quantum cryptography
• Instruction-level PQC optimization reduces cryptographic latency by 37% on edge devices
• RL-driven DSO-Engine adapts encryption, authentication, and privacy modes to real-time threats
• Achieves 42% higher energy efficiency and 99.88% availability on heterogeneous edge testbeds

## Graphical abstract

**Figure:**
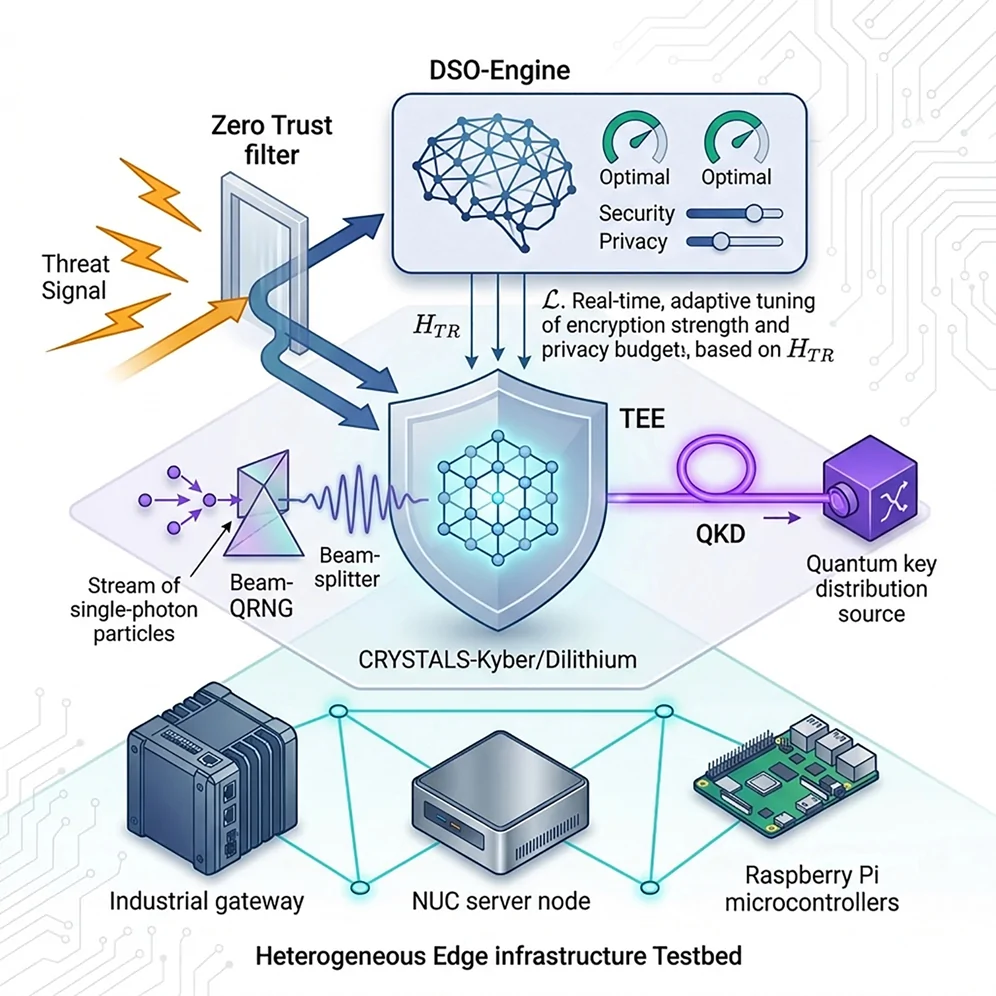

## Teaser

Computer science; Applied computing

## Introduction

The rapid convergence of the Internet of Things (IoT), artificial intelligence, and edge computing has catalyzed a fundamental shift in computing paradigms, wherein latency-sensitive and privacy-critical services are increasingly deployed at the network edge. By processing data proximate to its source, edge computing effectively reduces communication latency and bandwidth consumption while enabling real-time decision-making for applications ranging from autonomous vehicular networks to industrial IoT systems. However, this distributed architecture introduces inherent security vulnerabilities: edge nodes are geographically dispersed, physically accessible, resource-constrained, and exposed to highly dynamic workloads and threat conditions. These characteristics fundamentally undermine traditional perimeter-based security assumptions and render static security configurations ineffective in such heterogeneous environments.^1^^,^^2^

In response to these challenges, Zero Trust Architecture (ZTA)—grounded in the principle of “never trust, always verify”—has emerged as a foundational paradigm for edge and distributed systems, emphasizing continuous authentication, fine-grained access control, and strict isolation of sensitive computation.^3^ Simultaneously, the rapid advancement of quantum computing poses an existential threat to classical public-key cryptography, with widely deployed primitives such as RSA and elliptic curve cryptography expected to become vulnerable to polynomial-time quantum attacks. Consequently, next-generation edge security architectures must satisfy four stringent and often conflicting requirements: (1) quantum resistance to withstand cryptanalytic attacks by quantum adversaries; (2) computational and energy efficiency to accommodate resource-constrained edge devices; (3) dynamic adaptability to respond to rapidly changing threat landscapes and workload conditions; and (4) operational resilience to ensure sustained reliability across diverse hardware platforms and network topologies.

To address these multifaceted requirements, recent research has explored several complementary technical directions. Trusted Execution Environments (TEEs) have been widely adopted to protect sensitive code and data through hardware-assisted isolation, providing secure execution domains even under untrusted operating systems.^4^ Architectural enhancements have further strengthened TEE trust foundations; for instance, Hoang et al.^5^ proposed a heterogeneous TEE system-on-a-chip architecture that isolates the root-of-trust within a dedicated microcontroller-based subsystem, improving isolation strength and flexibility of secure boot and key management. Anasuri^6^ surveyed mainstream confidential computing frameworks including Intel SGX, Arm TrustZone, AMD SEV, and RISC-V Keystone, highlighting common challenges such as performance overhead, side-channel vulnerabilities, and scalability limitations in cloud and edge deployments. Recent advances have also extended trusted execution from process-level enclaves to virtual-machine-level and realm-based isolation, as exemplified by AMD SEV-SNP and Arm CCA, which improve protection against privileged software adversaries.^7^^,^^8^ Nevertheless, existing TEE-based solutions predominantly rely on classical entropy sources and traditional cryptographic primitives to establish roots of trust, leaving their security bounded by computational assumptions and vulnerable to quantum threats and prediction attacks.^9^^,^^10^

Concurrently, standardized post-quantum cryptographic (PQC) algorithms, particularly lattice-based schemes such as CRYSTALS-Kyber and Dilithium, offer algorithmic foundations for quantum-resistant communication.^11^ Comprehensive surveys have compared major PQC families across security assumptions, parameter sizes, and implementation complexity, while also synthesizing hybrid deployment strategies that balance quantum resistance with backward compatibility in heterogeneous networks.^12^^,^^13^ Hardware-oriented acceleration efforts have sought to reduce the cost of lattice-based operations, and hybrid classical-post-quantum frameworks demonstrate the practical necessity of combining existing mechanisms with post-quantum primitives.^14^^,^^15^ However, PQC algorithms incur substantially higher computational, memory, and energy overhead than classical cryptography, particularly on resource-constrained edge devices. Moreover, their execution within secure enclaves may exacerbate microarchitectural side-channel risks if not carefully optimized, yet existing optimization efforts have mainly focused on algorithm-level simplification or parameter tuning rather than enclave-aware execution characteristics.^16^^,^^17^

Furthermore, machine learning and reinforcement learning techniques have been introduced to dynamically adjust security policies in response to workload variations and threat dynamics.^18^^,^^19^ Reinforcement learning in particular has demonstrated strong potential for runtime security optimization by allowing agents to continuously learn from environmental feedback and adjust threat detection or mitigation strategies in real time.^20^ Complementing dynamic policy adaptation, privacy-preserving computing techniques—including federated learning, homomorphic encryption, and differential privacy—have been extensively explored to protect sensitive data during edge computation and distributed model training, though persistent challenges remain regarding computational overhead, convergence degradation, and vulnerability to inference attacks.^21^ Recent surveys emphasize that while machine learning-driven security frameworks improve adaptability and threat awareness in IoT and edge systems, most solutions rely on fixed privacy mechanisms and lack coordinated runtime switching across security, privacy, and system-performance dimensions.^22^ Recent studies on AI-enabled IoT and edge security have further advanced decentralized anomaly defense, federated graph-based DDoS detection, and self-evolving cognitive agents for adversarial environments.^23^^,^^24^^,^^25^^,^^26^ These contributions provide valuable advances in threat detection and adaptive defense, yet they primarily focus on anomaly detection or security decision support rather than embedding adaptive policy optimization into a quantum-safe trusted execution workflow.

Despite these advances, existing solutions predominantly treat TEE-based isolation, post-quantum cryptography, and adaptive security control as independent layers of the security stack, limiting their combined effectiveness in jointly achieving quantum security, performance efficiency, and operational adaptability. Specifically, current approaches exhibit three fundamental limitations. First, most TEE implementations rely on classical entropy sources and computational security assumptions for trust establishment, leaving their roots of trust vulnerable to quantum threats and prediction attacks.^9^^,^^10^ Second, lattice-based PQC algorithms incur substantial computational and memory overhead on resource-constrained edge devices, and their execution within secure enclaves may exacerbate microarchitectural side-channel risks if not carefully optimized.^16^^,^^17^ Third, existing security architectures predominantly adopt static or rule-based policies, lacking the ability to dynamically modulate encryption strength, access control granularity, and privacy-preserving computation modes in response to real-time threat intelligence and system load variations.^27^^,^^28^ Moreover, prior evaluations have largely focused on cryptographic microbenchmarks or short-term simulations, neglecting critical deployment factors such as heterogeneous device compatibility, network topology adaptability, and long-term operational stability—dimensions essential for production-grade edge systems.

To bridge these gaps, this paper presents Q-TEE-DSO as a cross-layer security orchestration framework for quantum-safe zero-trust edge computing. The framework builds on established security foundations, including trusted execution environments, lattice-based post-quantum cryptography, quantum-derived key support, and reinforcement-learning-based adaptive control, and organizes them into a unified runtime workflow. Its technical focus is the coordinated management of trust establishment, cryptographic execution, privacy-preserving computation, and policy adaptation under the joint constraints of latency, energy consumption, enclave execution, and heterogeneous edge deployment. As illustrated in Figure 1, Q-TEE-DSO integrates quantum random number generation (QRNG)/quantum key distribution (QKD)-assisted key support, enclave-aware PQC execution, and Dynamic Strategy Optimization Engine (DSO-Engine)-based policy optimization to enable adaptive security configuration in dynamic edge environments.

**Figure 1:**
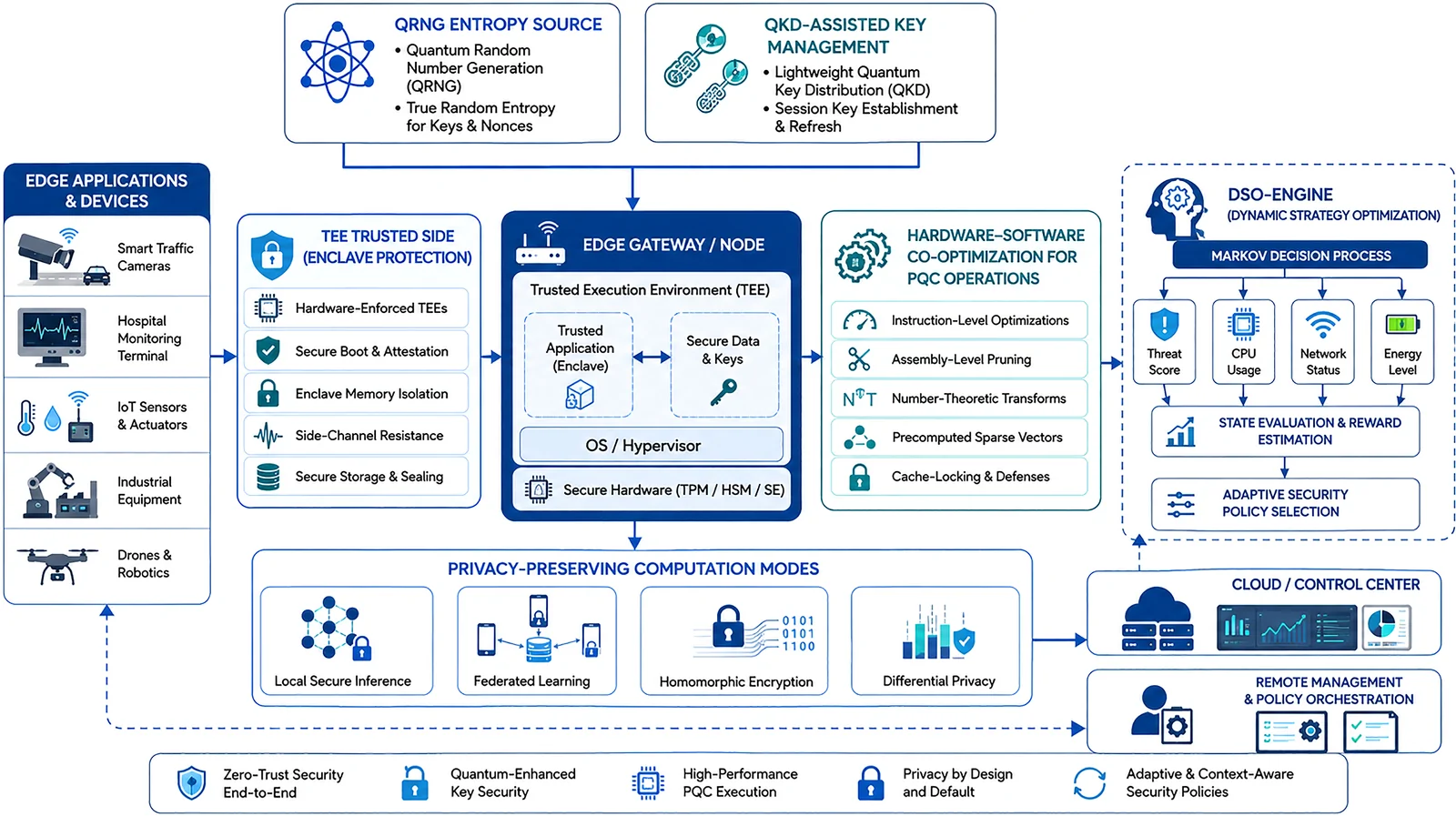
Quantum-enhanced trust root construction and key management architecture in Q-TEE-DSO The architecture integrates quantum random number generation (QRNG) and quantum key distribution (QKD)-assisted trust establishment, trusted execution environment (TEE)-protected post-quantum cryptographic (PQC) execution, and Dynamic Strategy Optimization Engine (DSO-Engine)-driven adaptive policy control. Cross-layer coordination among trust, cryptographic, privacy, and system-performance modules enables context-aware security configuration in heterogeneous edge environments.

The main system-level contributions of this work can be summarized as follows. First, we develop a cross-layer Q-TEE-DSO orchestration architecture that unifies the zero-trust edge security workflow by coordinating QRNG/QKD-assisted trust establishment, TEE-protected execution, optimized PQC operations, and adaptive policy control. This architecture enables security decisions to be made across trust, cryptographic, privacy, and system-performance layers rather than within isolated security modules. Second, we integrate instruction-level optimization, enclave-aware memory isolation, and side-channel-aware execution control for lattice-based PQC algorithms, reducing cryptographic latency and energy overhead on heterogeneous edge devices while incorporating leakage control into the trusted execution workflow. Third, we embed the DSO-Engine into the Q-TEE-DSO workflow to connect runtime threat assessment with adaptive selection of encryption strength, authentication mode, key-management policy, privacy budget, and privacy-preserving computation mode, thereby supporting context-aware security adaptation under changing workload and threat conditions. Fourth, we conduct an integrated evaluation of security-performance trade-offs across heterogeneous edge devices, network topologies, and long-duration operation scenarios, highlighting how coordinated security orchestration affects latency, energy efficiency, availability, and adaptive response in edge deployment settings.

## Results

### Experimental setup

To evaluate Q-TEE-DSO, we constructed a heterogeneous edge testbed comprising 10 Intel NUC minicomputers (Core i7-1165G7) as high-performance edge nodes, 15 Raspberry Pi 4 Model B units as resource-constrained end nodes, and an FPGA-based measurement-device-independent QKD system for quantum key generation. The NUC nodes ran Ubuntu 20.04 LTS with the Intel SGX SDK and DCAP driver, hosting the Q-TEE runtime with instruction-level optimized CRYSTALS-Kyber v3.0, Dilithium v3.1, and BFV homomorphic encryption libraries.

Runtime data were collected at 100 ms intervals by a low-overhead C++ probe deployed inside the Q-TEE enclave, recording CPU utilization, memory usage, network I/O, QKD key rate, and PQC operation latency. Application workloads were generated using a customized EdgeBench suite to simulate intelligent traffic monitoring and distributed health monitoring scenarios, with threat emulation (DoS attacks and port scans) performed via the Metasploit framework. Alert logs captured by Suricata, together with authentication failure rates, fed a simulated threat scoring engine that dynamically computed the ThreatScore for the DSO-Engine. The specific collected metrics and sampling rates are summarized in Table 1.

**Table 1:** Specific data items

Collected metrics	Definition and description	Sampling rate
CPU Utilization (%)	Average edge node processor utilization, reflecting computational load.	100 ms
Memory Usage (MB)	The physical memory size occupied by Q-TEE core components (including enclaves, DSO-Engine, and PQC libraries).	100 ms
Network I/O (KB/s)	The node’s network input/output bandwidth, used to measure communication overhead.	100 ms
QKD Key Generation Rate (bps)	The bit rate of the shared key successfully distributed by the QKD link for encryption.	1 s
PQC Encryption and Decryption Latency (ms)	The time required to perform a complete Kyber key encapsulation or decapsulation operation.	Event trigger (after each operation)
PQC Signature Verification Latency (ms)	The time required to perform a complete Dilithium signature generation and verification operation.	Event trigger (after each operation)

The implementation is organized into four reproducible components: system orchestration (key generation, encryption/decryption, secure session management, simulated QRNG/QKD support, and PQC interfaces), workload generation (synthetic EdgeBench-style traces), DSO-Engine training (DDPG agent, hyperparameters, and reward logging), and security-control pipeline demonstration. The released reproducibility package provides a sanitized lite implementation for architectural validation; physical QKD hardware operations, SGX enclave calls, and low-level side-channel mitigations are represented through software abstractions or simulated interfaces (see supplemental information for full details).

The enclave-side trusted computing base (TCB) encompassed only the Q-TEE trusted core, sealing/attestation interface, optimized Kyber/Dilithium routines, privacy-computing selector, and DSO inference interface, yielding a TCB size of 9.3 MB and a peak enclave memory usage of 58.7 ± 4.6 MB on NUC nodes. Raspberry Pi client-side trusted runtime footprint was 36.2 ± 3.1 MB. Cold-start latency—defined as the time from enclave creation and Q-TEE-DSO stack loading to the first valid secure policy decision—averaged 214.8 ± 18.5 ms on NUC nodes and 392.6 ± 34.7 ms on Raspberry Pi terminals, with overhead primarily attributable to enclave initialization, PQC library loading, sealing-key recovery, and DSO policy-state loading.

### Experimental evaluation

We compared five security configurations: (i) TEE+TLS1.3, using Intel SGX for local protection and TLS 1.3 with elliptic-curve key exchange for remote communication; (ii) PQC, running CRYSTALS-Kyber and Dilithium in normal memory without enclave isolation; (iii) Static Policy TEE, disabling the DSO-Engine and enforcing fixed parameters (Kyber-1024, Dilithium-5, BFV homomorphic encryption) across all nodes; (iv) RL-based Adaptive Edge Security, employing reinforcement learning for dynamic policy adjustment but relying on classical entropy and conventional key management; and (v) Proposed Method, the full Q-TEE-DSO architecture integrating QRNG/QKD-enhanced trust, optimized PQC, enclave-level side-channel mitigation, and DSO-Engine-driven adaptive policy control.

Experiments followed a controlled-variable methodology with 24-h stress-testing runs. An initial 1-h steady-state phase established baseline performance, after which dynamic scenarios were triggered via scripted events varying system load (50%–100%) and injecting cyber threats to elevate ThreatScore. QKD link conditions were adjusted in selected runs to reflect key availability variability. Runtime metrics were continuously collected by a centralized management server. Each experiment was repeated five times, and averaged results are reported. Statistical robustness was strengthened through standard deviations, 95% confidence intervals, and paired two-sided t-tests comparing the proposed method against the strongest directly comparable baseline under identical workload, device, and threat conditions. Significance is reported at *p* < 0.05, *p* < 0.01, and *p* < 0.001. The evaluation spans five application scenarios, three device categories, multiple network topologies, and long-duration traces.

To contextualize Q-TEE-DSO within the broader secure edge-computing landscape, we further compared its functional coverage against recent advances in confidential computing, PQC acceleration, hybrid classical-post-quantum security, AI-enabled IoT defense, and RL-based adaptive security. Given heterogeneity in platforms and deployment assumptions, this comparison emphasizes architectural scope rather than forced runtime equivalence. Table 2 summarizes these relationships.

**Table 2:** Extended comparison with recent secure edge-computing studies

Category	Representative studies	Main focus	Relation to Q-TEE-DSO
Confidential computing	SEV-SNP; Arm CCA	Isolation and attestation	Complementary layer
PQC acceleration	Kyber/Dilithium acceleration	Cryptographic speedup	Execution support
Hybrid post-quantum security	Hybrid framework; Q-AudioShield	Application protection	Scenario-specific
AI-enabled IoT defense	SwarmSense-DNN; GraphFedAI	Anomaly/DDoS detection	Threat-awareness support
RL-based adaptive security	PPO-GAN; NeuroAgent-X	Adaptive defense	Partial policy reference
Q-TEE-DSO	This study	Cross-layer orchestration	Integrated workflow

As shown in Table 2, existing confidential computing platforms primarily strengthen isolation and attestation, PQC acceleration studies reduce cryptographic execution cost, hybrid post-quantum frameworks target application-level protection, and AI/RL-based methods improve threat detection or adaptive defense. Q-TEE-DSO distinguishes itself by coordinating trusted execution, PQC execution, quantum-derived key support, privacy-preserving computation, and dynamic policy optimization within a unified zero-trust edge workflow.

### Privacy computing latency

Privacy computing latency is a critical metric for real-time edge processing. Table 3 reports average latency across five typical scenarios for all evaluated configurations.

**Table 3:** Performance differences among different solutions

Solution	Intelligent traffic monitoring (ms)	Distributed health monitoring (ms)	Edge data aggregation (ms)	Edge node authentication (ms)	Terminal data preprocessing (ms)
TEE+TLS1.3	185	198	175	182	168
PQC Solution	230	245	210	228	205
Static TEE	152	165	140	155	142
RL-based Adaptive Edge Security	145	158	132	148	136
Proposed Method	116	124	108	112	102

The proposed method achieved the lowest latency across all scenarios. In intelligent traffic monitoring, latency was 116.0 ± 4.2 ms, representing a 37.3% reduction (95% CI: 33.8%–40.7%, *p* < 0.001) compared with TEE+TLS1.3 (185.0 ± 7.4 ms). Relative to the strongest adaptive baseline, RL-based Adaptive Edge Security (145.0 ± 5.7 ms), the proposed method achieved an additional 20.0% reduction (*p* = 0.004). Similar significance was observed across distributed health monitoring (124 ms vs. 198 ms, *p* < 0.001) and remaining edge scenarios. The pure PQC solution exhibited the highest latency (∼230 ms) due to unoptimized lattice-based operations, whereas static TEE and RL-based policies provided intermediate improvements. These results indicate that learning-based adaptation alone is insufficient to minimize latency under strict security constraints; the combined effect of instruction-level PQC optimization within Q-TEE and dynamic privacy-preserving mode selection by the DSO-Engine is necessary to achieve substantial gains.

To verify that instruction-level PQC optimization does not introduce observable leakage, we performed a TVLA-style fixed-versus-random check on Kyber and Dilithium execution inside the Q-TEE enclave, focusing on secret-handling segments including sampling, NTT computation, encapsulation/decapsulation, and signing. Timing and power-proxy traces showed no persistent first-order leakage patterns, suggesting that the optimized routines successfully reduced secret-dependent execution variation.

Overall, as visually summarized in Figure 2, the proposed method consistently outperforms static and adaptive baselines in real-time responsiveness while preserving strong security guarantees, highlighting its suitability for latency-sensitive edge environments.

**Figure 2:**
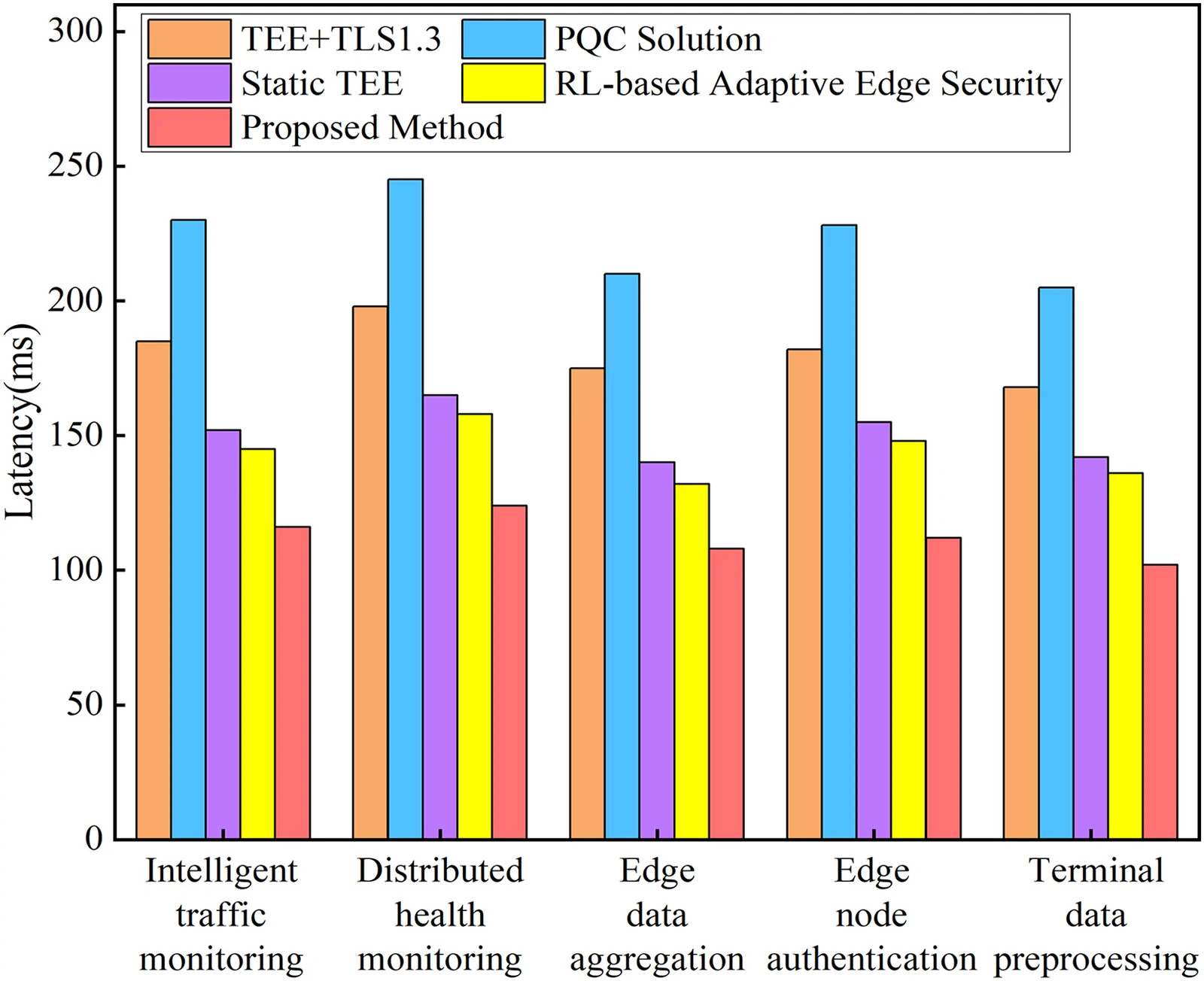
Comparison of privacy computing latency across different edge application scenarios The proposed method consistently achieves the lowest latency across all evaluated scenarios, including intelligent traffic monitoring, distributed health monitoring, edge data aggregation, node authentication, and terminal data preprocessing, outperforming both static configurations and learning-based adaptive baselines.

### Authentication energy efficiency

Authentication energy efficiency directly impacts battery life for resource-constrained edge devices. Table 4 summarizes energy efficiency (authentications/Wh) across heterogeneous device types and authentication frequencies.

**Table 4:** Comparison of energy efficiency data of different solutions

Solution	NUC edge node (1 time/second)	NUC edge node (10 times/second)	Raspberry Pi terminal (1 time/second)	Raspberry Pi terminal (10 times/second)	Edge gateway (1 time/second)	Edge gateway (10 time/second)
TEE+TLS1.3	85	72	60	48	90	78
PQC Solution	50	38	30	22	55	42
Static TEE	100	85	75	62	105	92
RL-based Adaptive Edge Security	108	95	80	66	112	98
Proposed Method	121	108	85	70	128	114

The proposed method achieved the highest energy efficiency across all configurations. On NUC edge nodes at 1 authentication/s, it reached 121.0 ± 5.8 authentications/Wh, a 42.4% improvement over TEE+TLS1.3 (85.0 ± 4.9, *p* < 0.001, 95% CI: 35.1%–49.6%) and a 12.0% improvement over RL-based Adaptive Edge Security (108.0 ± 4.7, *p* = 0.012). Similar gains persisted at 10 authentications/s (108 vs. 72 authentications/Wh on NUC) and across Raspberry Pi terminals (85 vs. 60 at 1/s; 70 vs. 48 at 10/s) and edge gateways (128 vs. 90 at 1/s; 114 vs. 78 at 10/s), confirming effectiveness under both low- and high-frequency workloads.

Energy decomposition revealed that PQC and TEE cryptographic execution accounted for the largest share (52.4%) of authentication energy consumption, followed by standard data processing and network I/O (22.1%), quantum-derived key support (18.7%), and DSO-Engine inference (6.8%). The efficiency gains primarily stem from the DSO-Engine’s dynamic selection of low-overhead authentication modes and the lightweight QKD-enabled key establishment, which jointly reduce unnecessary high-strength cryptographic execution and avoid static over-provisioning of privacy-computing modes. These results are visually summarized in Figure 3.

**Figure 3:**
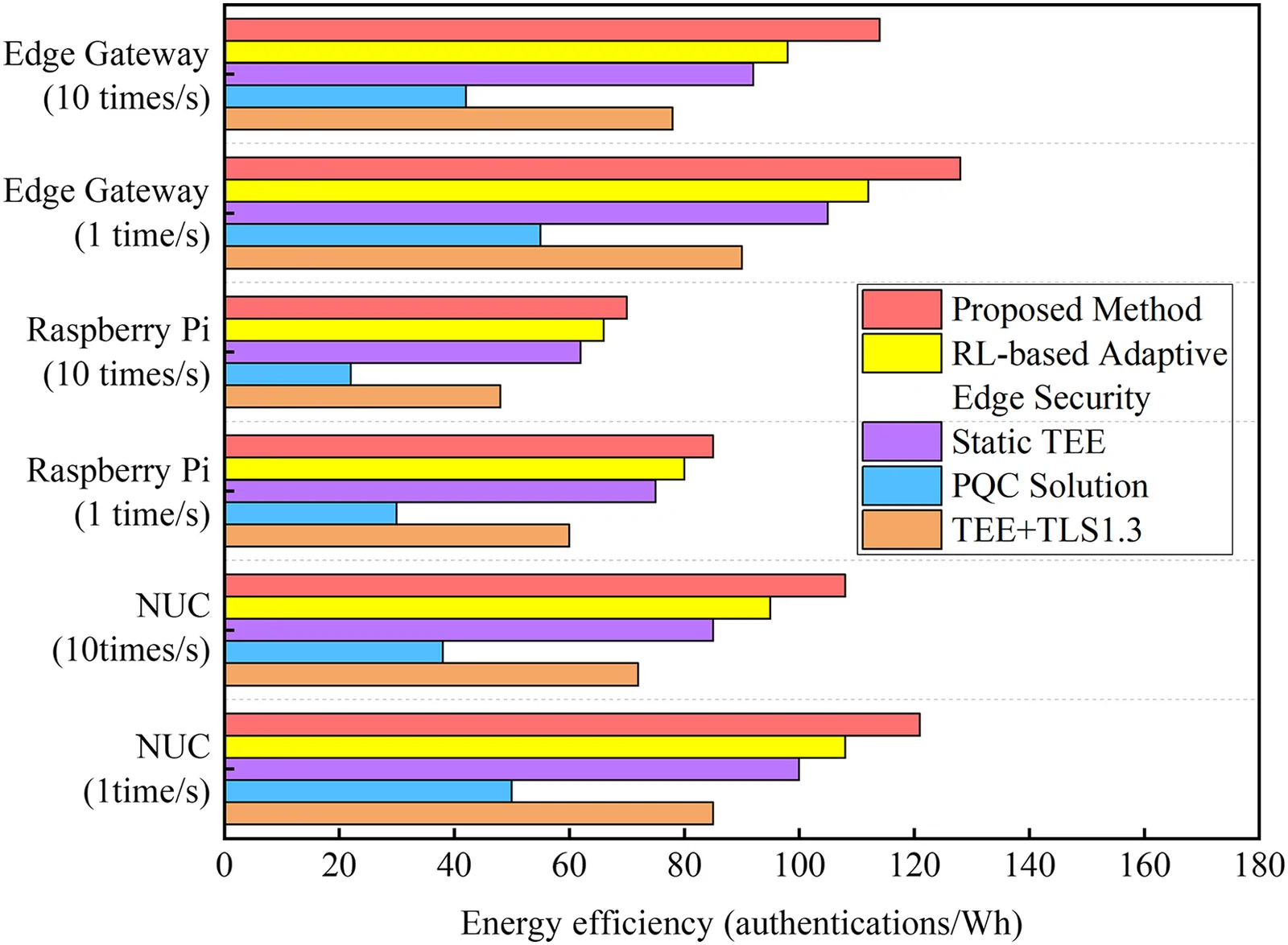
Authentication energy efficiency comparison across heterogeneous edge devices and authentication frequencies The proposed method consistently achieves the highest authentication energy efficiency across all device types and authentication frequencies, significantly outperforming classical, post-quantum, and learning-based adaptive baselines through coordinated QKD-enabled key establishment and DSO-Engine dynamic policy selection.

### Policy decision response time

Policy decision response time determines a dynamic security architecture’s ability to adapt to real-time threats and load fluctuations. Table 5 summarizes response times across typical event scenarios for solutions capable of dynamic adaptation (static policy TEEs are excluded due to the absence of dynamic decision-making capabilities).

**Table 5:** Response time in different scenarios

Solution	Threat level increase response (ms)	System load surge (ms)	Network fluctuation (ms)	Node access authentication (ms)	Key update (ms)
TEE+TLS1.3	250	230	280	260	240
PQC Solution	300	280	320	310	290
RL-based Adaptive Edge Security	170	150	190	175	160
Proposed Method	120	100	150	130	110

The proposed method consistently maintained response latency below 150 ms, achieving an average of 122.4 ± 6.3 ms under dynamic threat and workload variation. This corresponds to 48.4% of the response time observed under TEE+TLS1.3 and a 28.2% improvement over RL-based Adaptive Edge Security, with paired comparisons showing statistical significance (*p* < 0.01). The fastest response (100 ms) occurred during system load surges, benefiting from the DSO-Engine’s fine-grained resource monitoring and rapid policy selection. Network fluctuation and key update scenarios exhibited relatively higher latency (150 ms and 110 ms, respectively) due to additional coordination between policy decisions and security state adjustments; nevertheless, all scenarios remained within real-time operational requirements. These results are illustrated in Figure 4.

**Figure 4:**
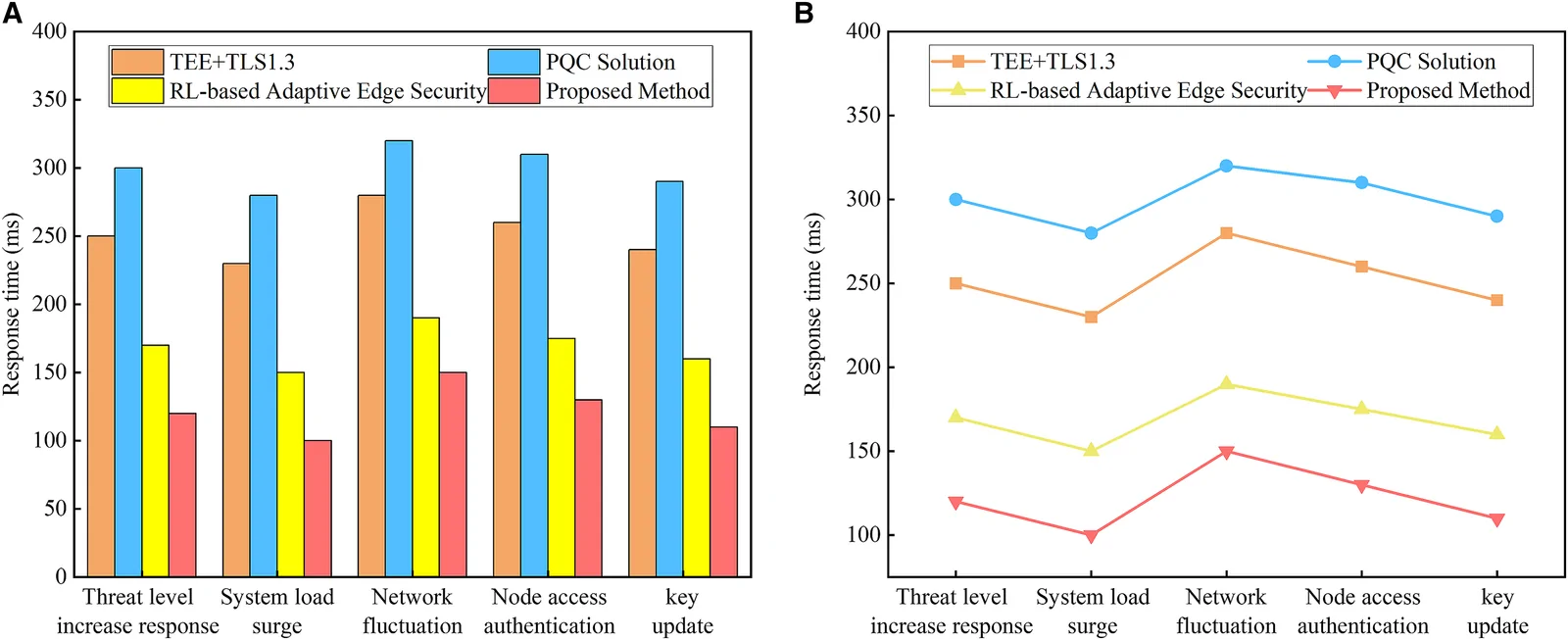
Policy decision response time under different event scenarios The proposed method maintains the lowest policy decision response time across all evaluated event scenarios, enabling rapid adaptive security configuration under dynamic threat and workload conditions.

### Ablation and interpretability analysis of the DSO-Engine

In addition to the ablation comparison, we evaluated the robustness of the DSO-Engine under abnormal input conditions, including ThreatScore amplification and suppression, QKD key-rate collapse, burst packet loss, and load surges accompanied by alerts; the results are summarized in Table 6. To isolate the specific contribution of the DSO-Engine, we compared four policy-management strategies under identical workload traces and threat-injection settings: a static policy with fixed encryption, authentication, and privacy-preserving modes; a rule-based heuristic with predefined ThreatScore and load thresholds; a DDPG-only policy without safe-action filtering; and the full DSO-Engine combining DDPG output with safe-action constraints and zero-trust validation (Table 7).

**Table 6:** Robustness evaluation of the DSO-Engine under abnormal inputs

Abnormal condition	Perturbation setting	Evaluation focus	Policy behavior
ThreatScore amplification	Sudden increase in reported threat level	Switching stability	Higher security level
ThreatScore suppression	Partial masking of alert indicators	Downgrade prevention	Minimum security preserved
QKD key-rate collapse	Key rate drops below usable threshold	Key-management robustness	PQC fallback activated
Burst packet loss	Sharp increase in packet loss	Authentication robustness	Stronger authentication
Load surge with alerts	High load with IDS alert increase	Security-performance balance	Conservative configuration

**Table 7:** Ablation analysis of the DSO-Engine

Policy strategy	Policy mechanism	Latency behavior	Security adaptation	Policy stability	Overall observation
Static policy	Fixed configuration	Stable but less efficient	Low	High	Cannot respond to threat changes
Rule-based heuristic policy	Threshold-triggered switching	Moderate	Moderate	Moderate	Sensitive to threshold design
DDPG without safe-action filter	Learned policy only	Low	Moderate	Lower	May produce invalid actions
Full DSO-Engine	Learned policy with safe-action filter	Low	High	High	Best security-performance balance

The static policy exhibited high stability but failed to adapt to changing threats or load. The rule-based heuristic improved adaptability yet remained sensitive to manually tuned thresholds, causing frequent switching near boundary conditions. The DDPG-only strategy reduced latency through learned adaptation, but unconstrained outputs occasionally violated cryptographic or privacy constraints. The full DSO-Engine achieved the optimal balance by coupling learning-based optimization with security-constraint validation, enabling dynamic adaptation while preserving baseline zero-trust requirements.

To examine policy interpretability, we visualized the trained DSO-Engine’s decision boundaries in a two-dimensional map using ThreatScore and resource pressure as input dimensions (other state variables fixed at mean values), as shown in Figure 5. The DSO-Engine selected lightweight configurations under low ThreatScore and low resource pressure, progressively shifting toward stronger authentication, higher PQC parameter levels, and stricter privacy-preserving modes as ThreatScore increased. Under high resource pressure with low ThreatScore, the policy avoided unnecessarily expensive configurations; under concurrent high ThreatScore and high resource pressure, it selected conservative configurations that preserved security while limiting mode oscillation. This structured transition pattern confirms that the DSO-Engine learns interpretable, context-aware policy mappings rather than arbitrary outputs.

**Figure 5:**
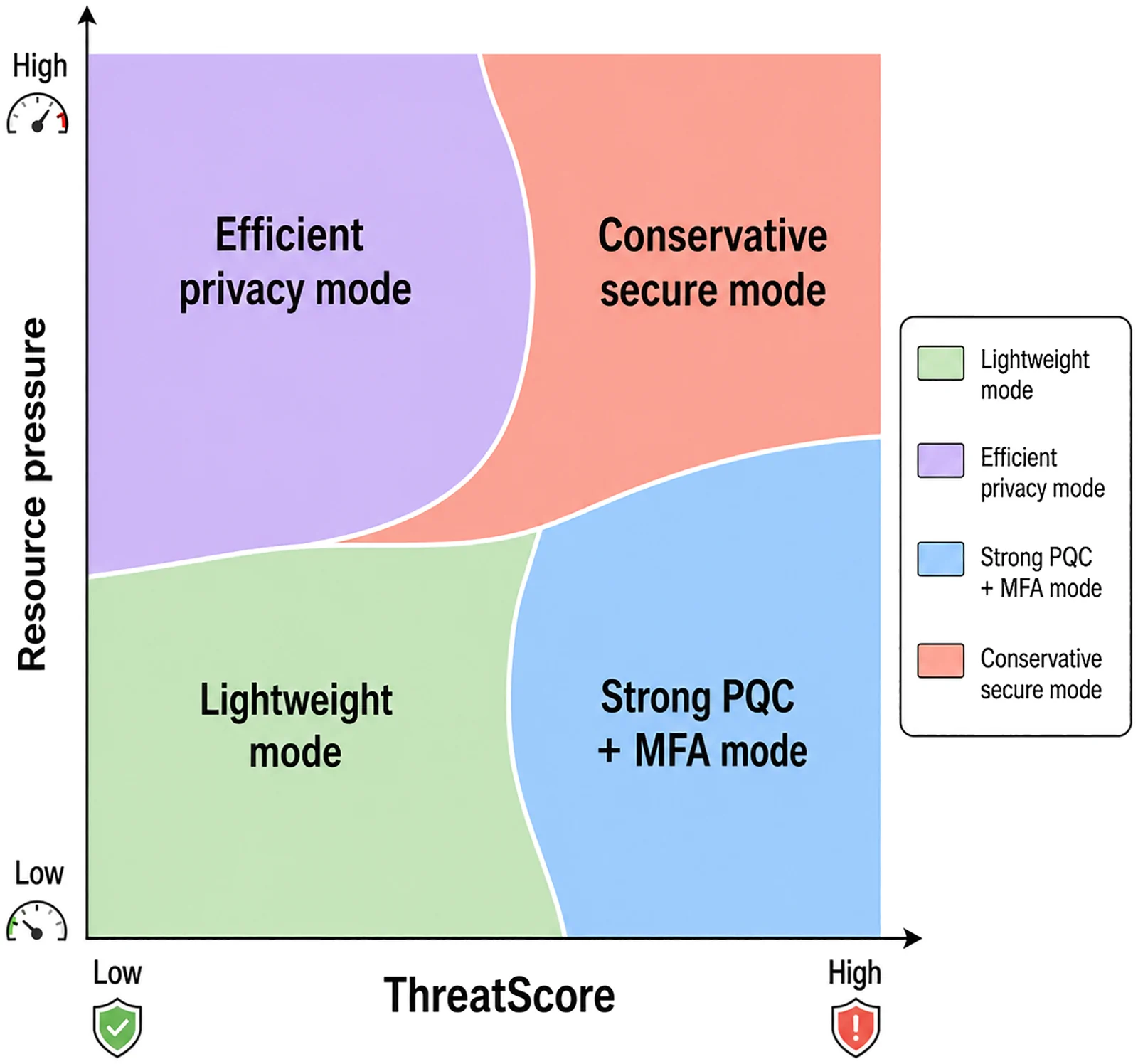
Decision-boundary visualization of the DSO-Engine policy The trained DSO-engine learns a structured policy transition pattern that selects lightweight configurations under low threat and resource pressure while shifting toward conservative security modes under high-risk conditions.

### Quantum security evaluation

We evaluated quantum security under a composite adversarial model combining quantum-accelerated key search and quantum-assisted side-channel amplification. Quantum attack intensity (*I*) was defined as a normalized indicator of the adversary’s effective quantum computational and leakage-extraction capability, with four representative levels: *I* = 2 (low), *I* = 4 (medium), *I* = 6 (high), and *I* = 8 (extreme). Table 8 reports key survival rate and key regeneration latency across all configurations.

**Table 8:** Security performance under different quantum attack intensities

Attack intensity	Method	Key survival rate (%)	Key regeneration latency (ms)
Low (I = 2)	TEE + TLS1.3	72	250
	PQC Solution	80	280
	RL-based Adaptive Edge Security	84	170
	Proposed Method	95	110
Medium (I = 4)	TEE + TLS1.3	48	260
	PQC Solution	65	310
	RL-based Adaptive Edge Security	70	180
	Proposed Method	88	120
High (I = 6)	TEE + TLS1.3	18	300
	PQC Solution	42	350
	RL-based Adaptive Edge Security	50	210
	Proposed Method	72	135
Extreme (I = 8)	TEE + TLS1.3	8	340
	PQC Solution	30	390
	RL-based Adaptive Edge Security	38	240
	Proposed Method	55	155

The proposed method consistently outperformed all baselines across attack intensities while maintaining the lowest key regeneration latency. At low intensity (*I* = 2), it achieved a 95% key survival rate compared with 72% for TEE+TLS1.3, 80% for PQC, and 84% for RL-based Adaptive Edge Security. As intensity escalated to *I* = 6, the survival rate of TEE+TLS1.3 collapsed to 18%, whereas the proposed method retained 72%—a 4-fold improvement over classical cryptography and substantially exceeding PQC (42%) and RL-based (50%) solutions. Even under extreme quantum attacks (*I* = 8), the proposed method preserved the highest survival rate at 55%, while all baselines fell below 40%. Concurrently, key regeneration latency remained stable at 110–155 ms for the proposed method, compared with 250–340 ms for TEE+TLS1.3 and 280–390 ms for standalone PQC. These results demonstrate that the integration of quantum-enhanced key management with adaptive policy orchestration achieves superior quantum resilience without sacrificing system responsiveness, as visually summarized in Figure 6.

**Figure 6:**
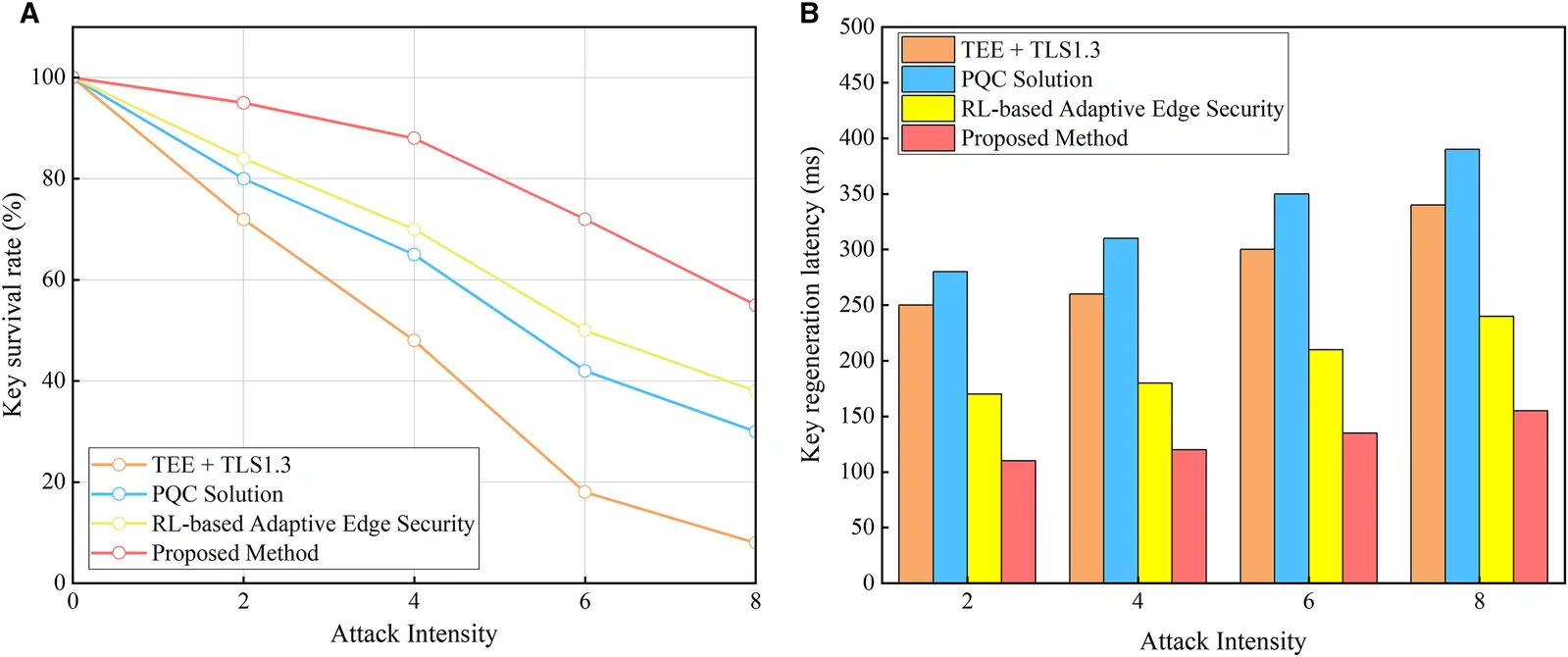
Quantum security performance comparison under different attack intensities The proposed method maintains the highest key survival rate and lowest regeneration latency across all quantum attack intensities, demonstrating superior quantum resilience through integrated QKD-enhanced trust and adaptive PQC execution.

### System load adaptability

We evaluated system throughput and CPU utilization under workload levels increasing from 40% to near-saturation (100%) to assess load adaptability (Table 9).

**Table 9:** System throughput and CPU utilization under different system loads

System load (%)	Method	Throughput (Mbps)	CPU utilization (%)
40	TEE + TLS1.3	85	40
	PQC only	70	50
	Static TEE	88	45
	RL-based Adaptive Edge Security	82	42
	Proposed Method	90	35
60	TEE + TLS1.3	100	62
	PQC only	85	78
	Static TEE	105	72
	RL-based Adaptive Edge Security	98	60
	Proposed Method	110	58
80	TEE + TLS1.3	95	80
	PQC only	75	90
	Static TEE	100	85
	RL-based Adaptive Edge Security	92	78
	Proposed Method	120	75
100	TEE + TLS1.3	85	90
	PQC only	65	94
	Static TEE	90	92
	RL-based Adaptive Edge Security	88	85
	Proposed Method	125	82

The proposed method consistently achieved the highest throughput and lowest CPU utilization across all load levels. At 40% load, it delivered 90 Mbps at 35% CPU utilization, compared with 85 Mbps/40% for TEE+TLS1.3 and 70 Mbps/50% for PQC-only. As load approached 100%, all baselines exhibited degradation or saturation: TEE+TLS1.3 throughput dropped to 85 Mbps at 90% CPU, PQC-only fell to 65 Mbps at 94% CPU, and Static TEE plateaued at 90 Mbps/92% CPU. In contrast, the proposed method scaled to 125 Mbps at 82% CPU. The RL-based adaptive baseline provided moderate improvements over static configurations but remained constrained under heavy load (88 Mbps/85% CPU at 100%). These results demonstrate that the DSO-Engine’s adaptive orchestration effectively mitigates performance degradation under saturation conditions without excessive resource consumption, as illustrated in Figure 7.

**Figure 7:**
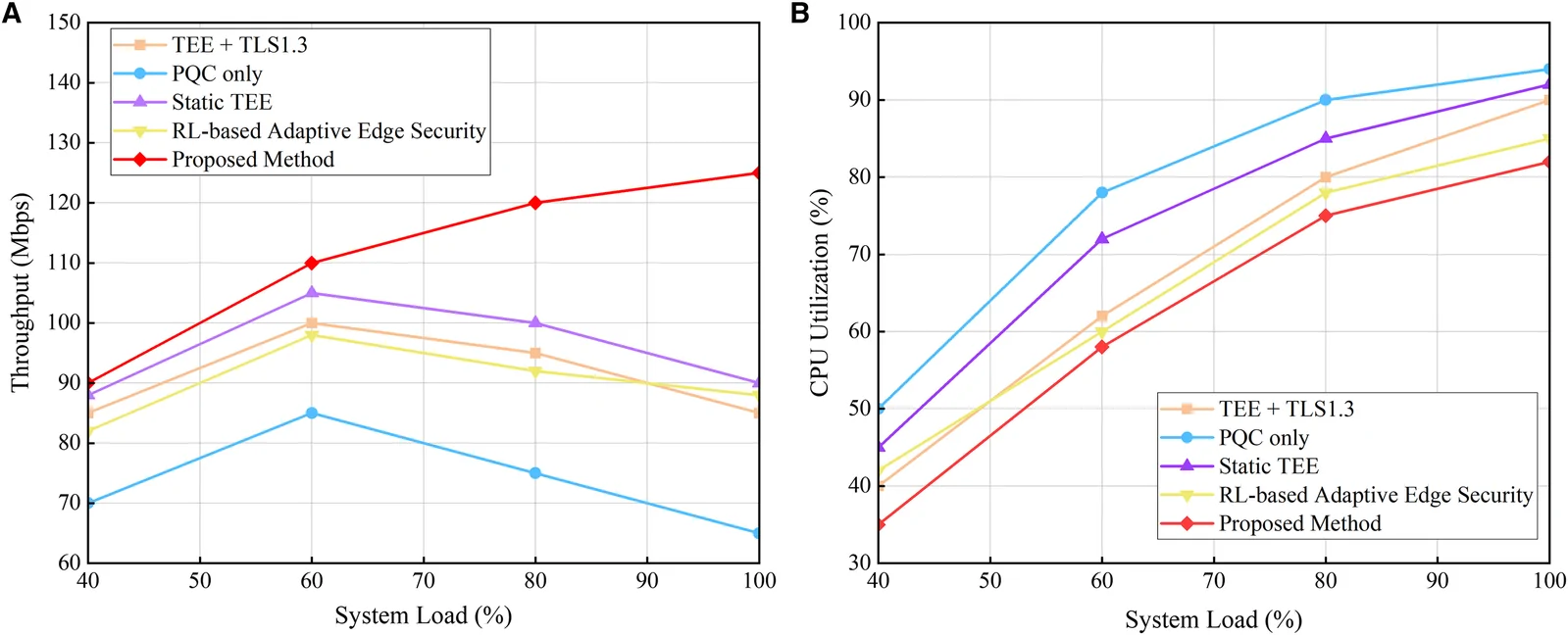
System throughput and CPU utilization under different system loads The proposed method achieves superior load adaptability by maintaining the highest throughput and lowest CPU utilization across all workload levels from moderate to near-saturation states.

### Heterogeneous device compatibility

To assess cross-platform deployability, we evaluated the proposed method across high-performance NUC nodes (i3, i5, i7), resource-constrained Raspberry Pi terminals (3B+, 4B), and edge gateways, measuring latency, throughput, CPU utilization, memory consumption, and energy efficiency. As shown in Figure 8, the proposed method maintained sub-150 ms latency even on lower-end NUC i3 (145 ms) and Raspberry Pi 3B+ (158 ms), while edge gateways achieved 92–125 Mbps throughput depending on hardware specifications. CPU utilization ranged from 35% to 55% on NUC nodes to 58%–85% on Raspberry Pi devices, reflecting the computational intensity of PQC operations on constrained hardware. Memory consumption scaled efficiently across platforms, from 145 MB on NUC i7 to 245 MB on Raspberry Pi 3B+. Energy efficiency reached 121 authentications/Wh on NUC i7. A holistic compatibility radar chart confirmed security scores above 0.95 across both NUC and Raspberry Pi platforms, with system startup times ranging from 2.5 s (NUC i7) to 7.2 s (Pi 3B+). These results demonstrate that instruction-level optimizations and adaptive resource management enable efficient execution across the performance spectrum from high-end edge nodes to resource-constrained terminals, validating the practical deployability of the Q-TEE architecture in diverse edge computing environments.

**Figure 8:**
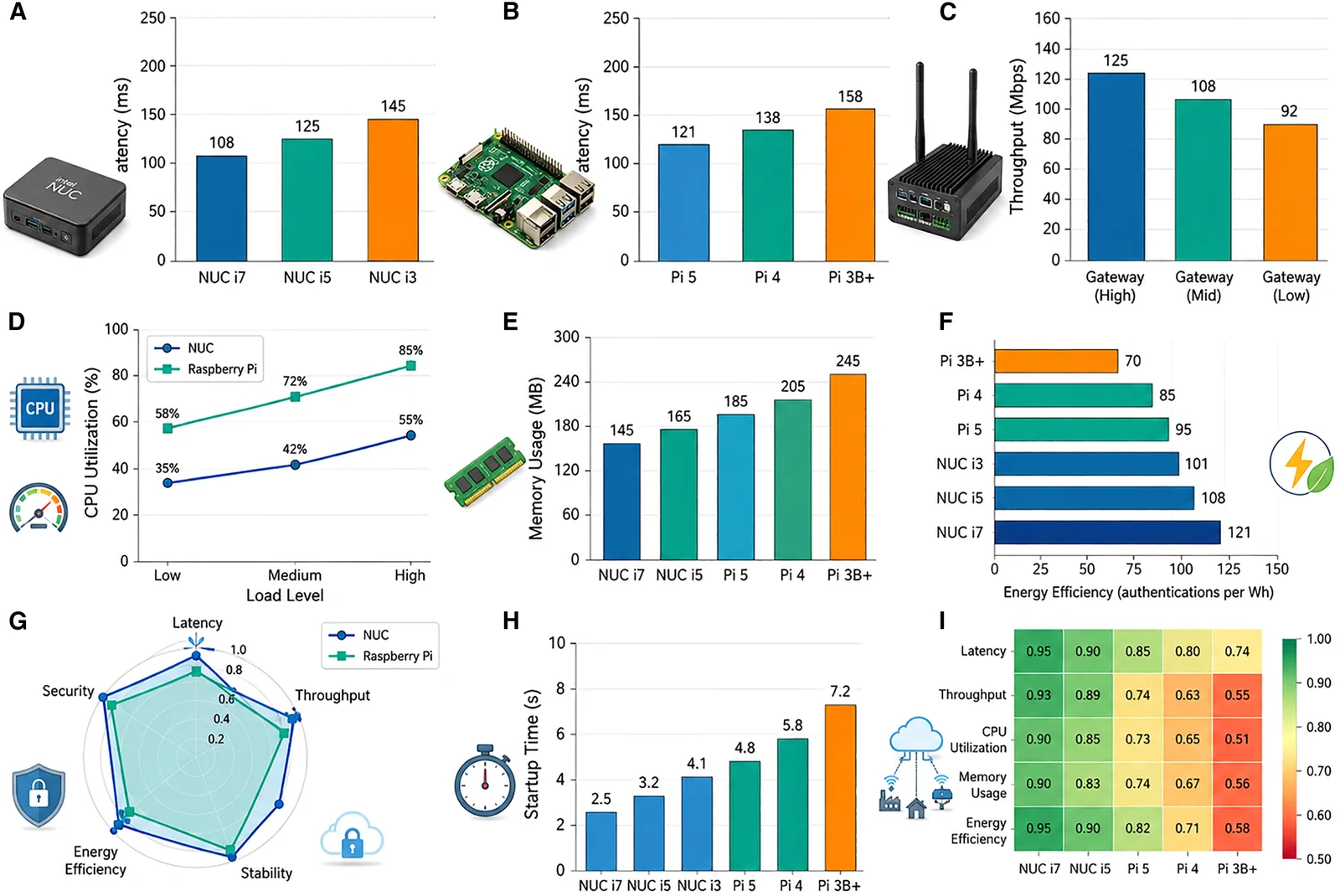
Heterogeneous device compatibility evaluation The proposed architecture maintains consistent security guarantees and efficient execution across diverse hardware configurations from high-performance NUC nodes to resource-constrained Raspberry Pi terminals.

### Network topology adaptability

We evaluated the proposed architecture across star, mesh, tree, ring, and hybrid topologies, examining latency, throughput, scalability, and fault tolerance (Figure 9). The mesh topology achieved the lowest average latency (107 ms) and highest throughput (112–145 Mbps), outperforming hybrid (112 ms), star (118 ms), tree (128 ms), and ring (135 ms) configurations. Mesh topology also exhibited superior path redundancy, maintaining stable latency across increasing hop counts (88–115 ms for 1–5 hops) and scaling efficiently from 5 to 100 nodes (88–145 ms), whereas star topology degraded from 95 ms to 215 ms. Bandwidth utilization remained high (75%–92%) across all configurations.

**Figure 9:**
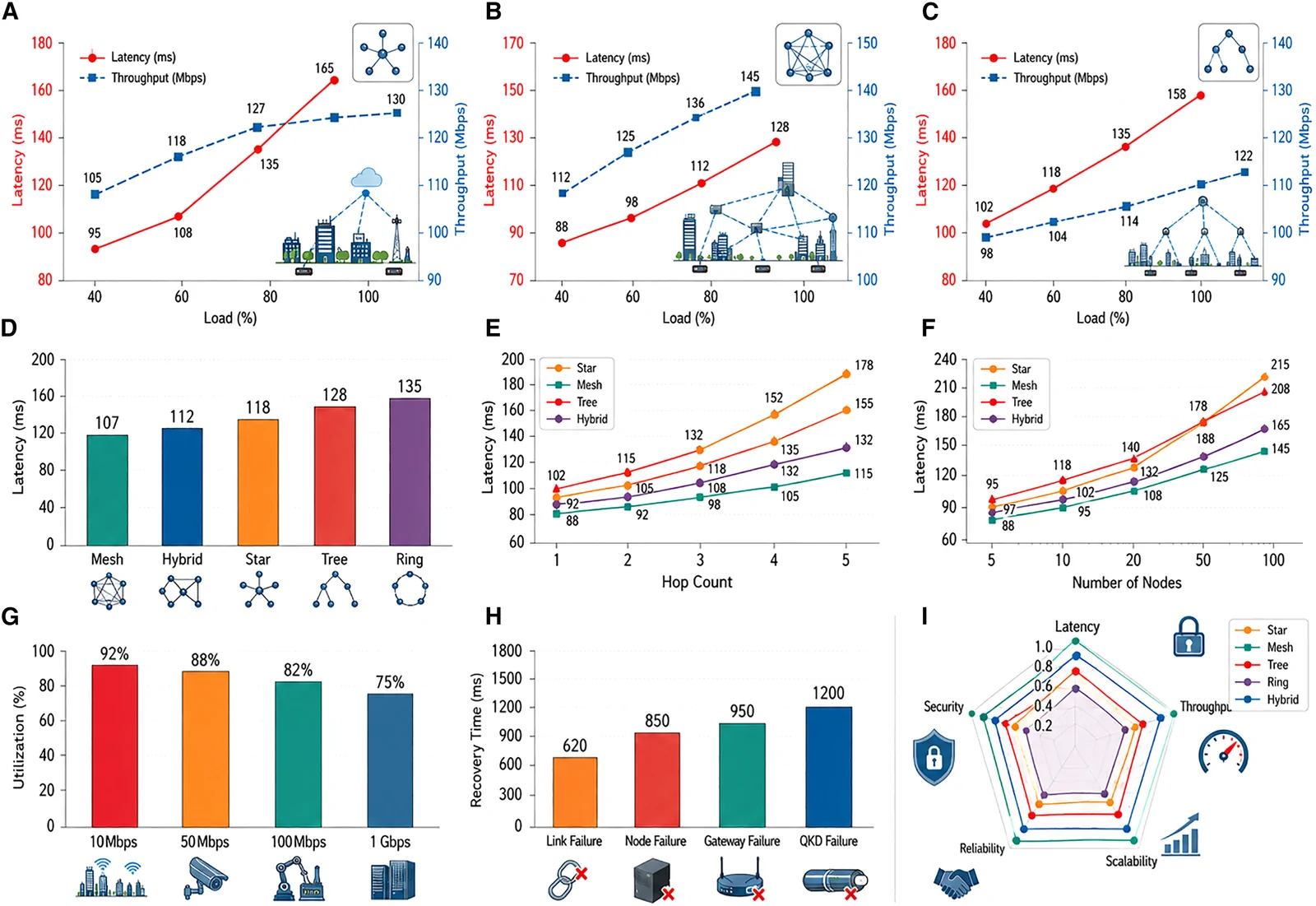
Network topology adaptability analysis The proposed architecture exhibits robust performance and scalability across diverse topological configurations, with mesh topology demonstrating superior latency stability and fault recovery characteristics for large-scale edge deployments.

To assess QKD key-refresh feasibility at scale, we simulated a smart-city deployment exceeding 500 nodes with gateway-level QKD resources and staggered refresh scheduling. QKD-assisted refresh remained a controlled fraction of backhaul load under normal intervals, while aggressive refresh triggered PQC fallback, confirming that gateway aggregation and scheduled coordination are essential for large-scale operation.

Fault recovery times were within acceptable limits: link failures (620 ms), node failures (850 ms), gateway failures (950 ms), and QKD failures (1,200 ms). The mesh topology achieved the highest overall scores, particularly in scalability (0.90) and reliability (0.95), while maintaining competitive security (0.85). These results demonstrate that the DSO-Engine’s dynamic policy optimization effectively compensates for topological constraints, enabling consistent security guarantees across diverse network infrastructures.

### Long-term stability and reliability

To assess production-readiness, we conducted a 7-day continuous operation test evaluating sustained performance, resource stability, error rates, and system reliability (Figure 10). Throughput remained stable around 119.8 Mbps with minor diurnal fluctuations. CPU utilization averaged 65% (occasional peaks at 75%), while memory leak rate was minimal at 2.5 MB per hour (total growth from 512 MB to ∼930 MB over 168 h). QKD key generation maintained an average of 1,000 bps (range: 800–1,100 bps), consistently meeting the 900 bps minimum requirement. Authentication success rates exceeded 99.6% throughout the test period, with cumulative failures below 0.4%. Error distribution over 7 days totaled 43 incidents: network errors (15), timeouts (12), authentication failures (8), QKD errors (5), and PQC errors (3).

**Figure 10:**
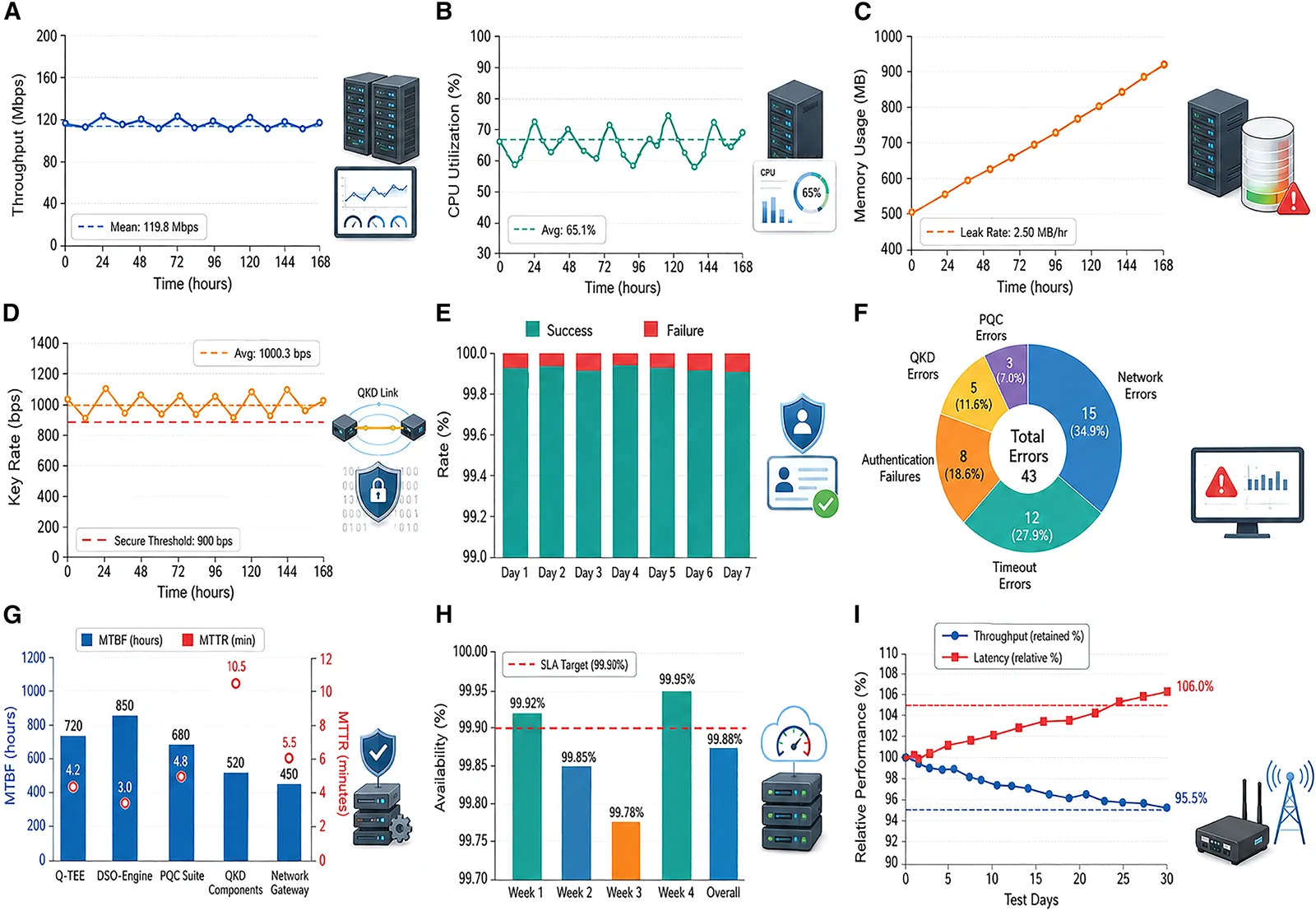
Long-term stability and reliability testing The proposed architecture maintains consistent performance, minimal resource degradation, and high availability over extended operational periods, validating its suitability for production-grade edge computing environments.

Reliability metrics demonstrated robust operational characteristics. The DSO-Engine achieved the highest component reliability with an MTBF of 850 h and MTTR of 3 min, while QKD components exhibited the lowest MTBF at 520 h. System availability consistently met the 99.9% SLA target, reaching 99.88% overall and improving to 99.95% by week 4. Over 30 days of continuous operation, throughput degraded minimally from 100% to 95.5% of baseline, while latency increased to 106%—both within acceptable limits for long-term deployment.

These results validate that the integration of quantum-enhanced trust mechanisms, optimized post-quantum cryptography, and adaptive policy control achieves both security robustness and operational stability required for mission-critical edge computing environments.

## Discussion

This study presents Q-TEE-DSO, a cross-layer framework that co-designs quantum-enhanced trust establishment, instruction-level optimized post-quantum cryptography, and reinforcement learning-driven adaptive control. Unlike prior approaches treating these components as independent layers, our results demonstrate that meaningful performance and security gains emerge only through holistic optimization. Integrating QRNG and lightweight QKD within the TEE trust root substantially enhances resilience against quantum-capable adversaries, yielding key survival rates of 95% under low-intensity and 55% under extreme quantum attacks. This outperforms purely computational security while maintaining key regeneration latency below 155 ms even under extreme conditions. The DSO-Engine’s dynamic modulation of encryption strength and privacy budgets represents a shift from static “always-maximum” security to risk-proportional protection, reducing unnecessary overhead during low-threat periods while ensuring cryptographic agility during attacks. The detailed training settings and reproducibility configuration of the DSO-Engine are summarized in Table 10. However, this adaptation depends on threat intelligence accuracy and system state granularity. Our current ThreatScore relies on IDS alerts and authentication failures; future work should incorporate quantum-specific indicators and cross-node behavioral correlation. Security claims are bounded by the stated threat model (Table 11): symbolic analysis verifies protocol-level key secrecy, authentication, replay resistance, and session binding (Table 12), but does not prove absence of microarchitectural leakage, physical tampering, compromised quantum hardware, or supply-chain risks. The detailed designs of these components are presented in the STAR Methods, including the QRNG hardware architecture (Figure 11), the lightweight BB84 quantum key distribution protocol (Figure 12), and the DDPG training convergence of the DSO-Engine (Figure 13).

**Table 10:** Key reproducibility settings of the DSO-Engine

Item	Configuration
State inputs	CPU utilization, memory usage, network throughput, QKD key rate, PQC latency, IDS alerts, authentication failures, and policy-switching history
Action outputs	Encryption level, authentication mode, key-refresh mode, privacy budget, and privacy-preserving computation mode
Actor network	Two fully connected hidden layers with ReLU activation
Critic network	State–action network with two fully connected hidden layers and ReLU activation
Optimizer	Adam
Learning rates	Actor: 1e−4; Critic: 1e−3
Discount factor and soft update	*γ* = 0.99; *τ* = 0.005
Training batch and memory	Mini-batch size: 64; replay memory: 100000 transitions
Exploration strategy	Action noise with standard deviation of 0.1
Security configuration range	Kyber-512/768/1024, Dilithium levels 2/3/5, 256-bit simulated QKD key, and privacy-budget adjustment

**Table 11:** Threat model and security scope of Q-TEE-DSO

Threat type	Adversary capability	Security mechanism
Network attack	Eavesdropping, replay, MITM	PQC handshake, nonce, session binding
Privileged software attack	OS or hypervisor compromise	TEE isolation, sealed key handling
Node impersonation	Fake edge identity	Remote attestation, signature check
Quantum-capable attack	Long-term cryptanalytic threat	Kyber/Dilithium, QKD-assisted keying
Side-channel observation	Timing/cache leakage attempt	Cache-aware control, empirical testing
QKD disruption	Key-rate drop or channel failure	PQC fallback
Runtime manipulation	Abnormal ThreatScore input	Safe-action filter
Out-of-scope attack	Hardware Trojans, physical extraction	Deployment-level control

**Table 12:** Symbolic security properties of Q-TEE-DSO

Property	Verification target	Result
Session-key secrecy	Session key not derived by attacker	Verified
Mutual authentication	Device and enclave agreement	Verified
Replay resistance	Old sessions rejected	Verified
MITM resistance	Session parameters protected	Verified
QKD fallback continuity	PQC fallback maintains setup	Verified
Side-channel resistance	Not modeled symbolically	Evaluated separately

**Figure 11:**
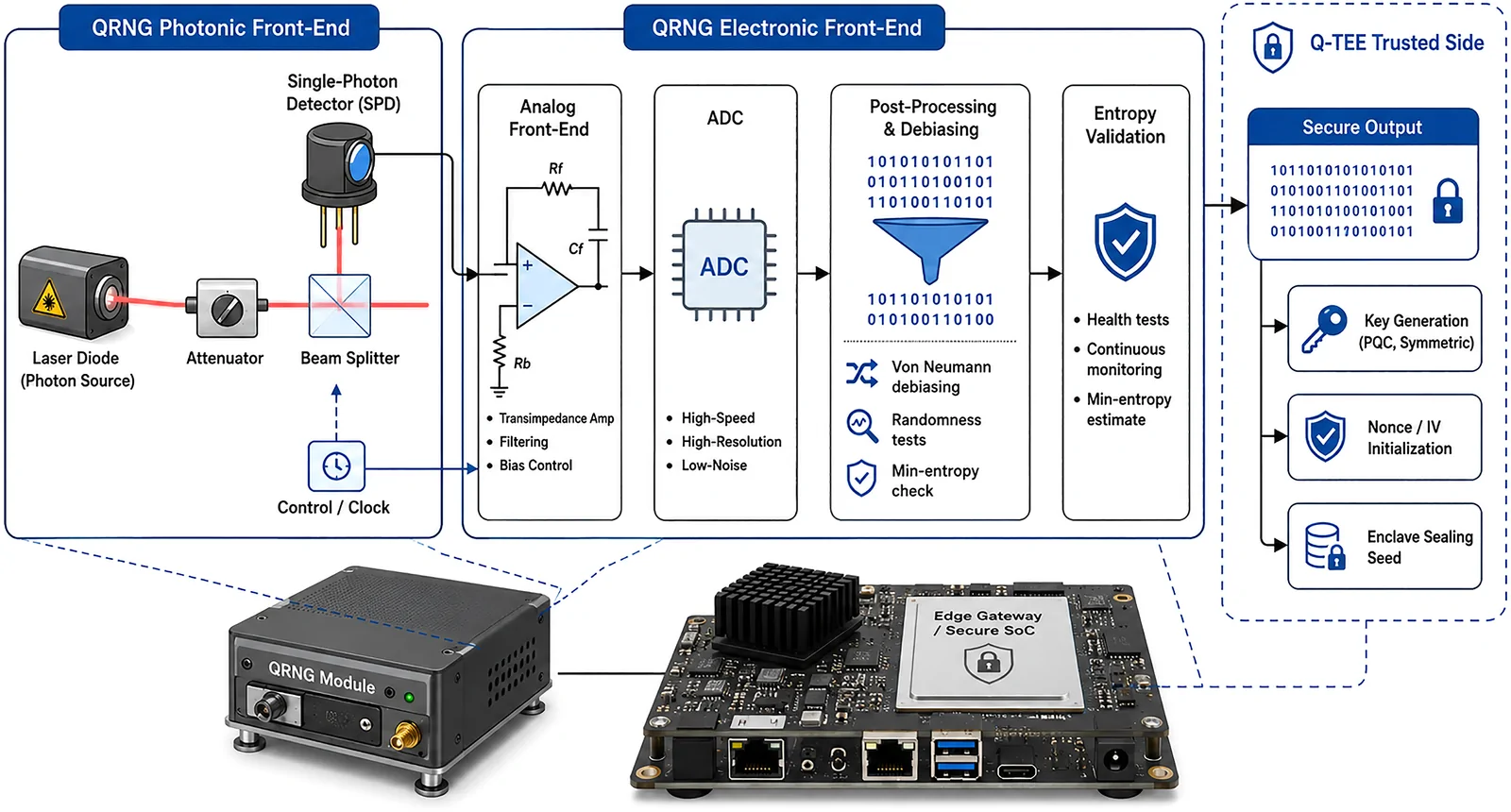
QRNG hardware architecture for quantum-enhanced trust root The architecture incorporates entropy validation, debiasing, and min-entropy extraction to generate unpredictable seeds for post-quantum key generation, nonce initialization, and enclave sealing under non-ideal edge conditions.

**Figure 12:**
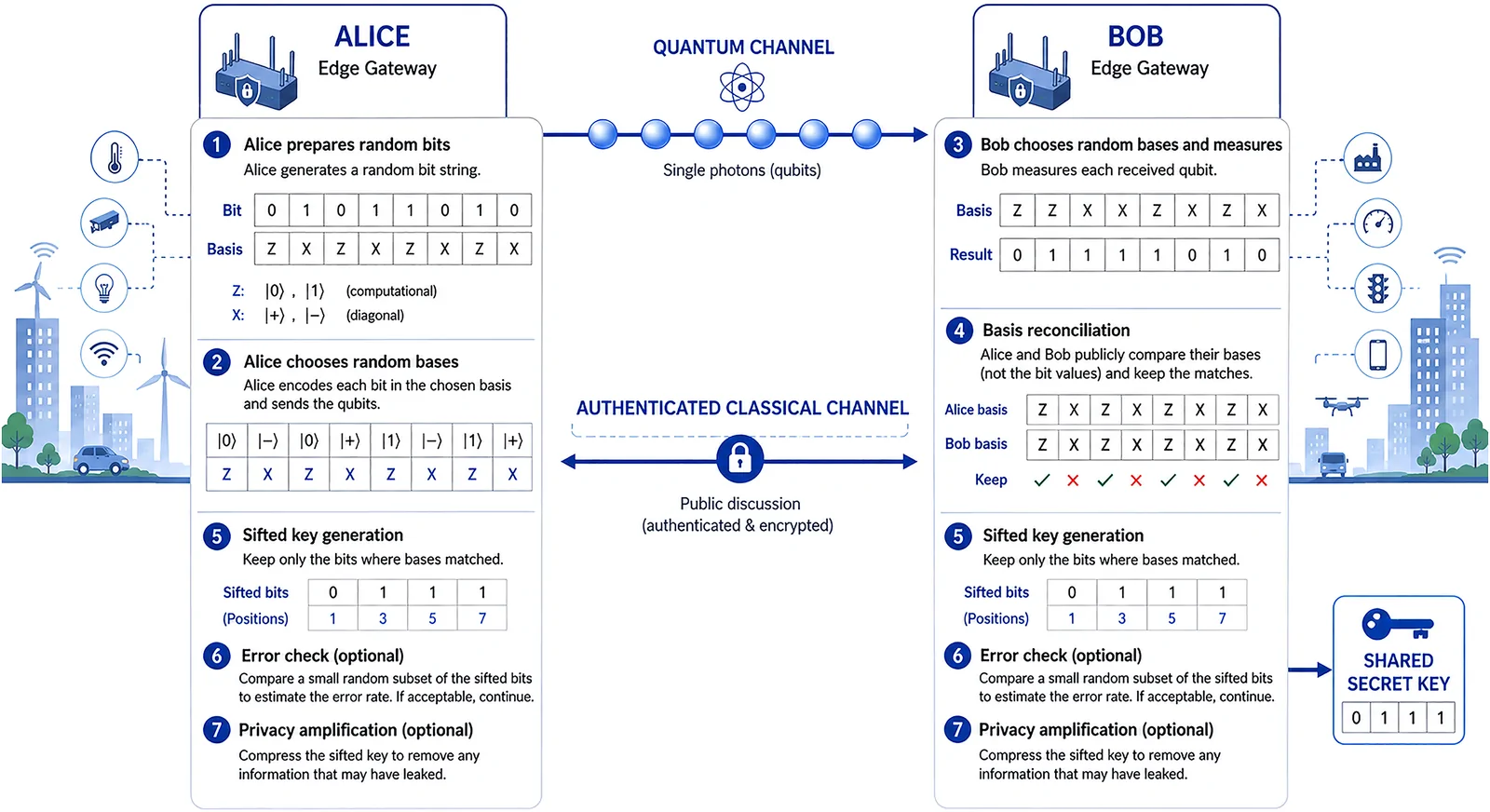
Lightweight BB84 quantum key distribution protocol for hybrid edge deployment The protocol illustrates quantum state preparation, basis selection, and measurement between the QKD transmitter (Alice) and receiver (Bob) over quantum and authenticated classical channels. Deployment is constrained to gateway-level or high-value edge links, with PQC-based key encapsulation serving as the fallback path for QKD-unavailable devices.

**Figure 13:**
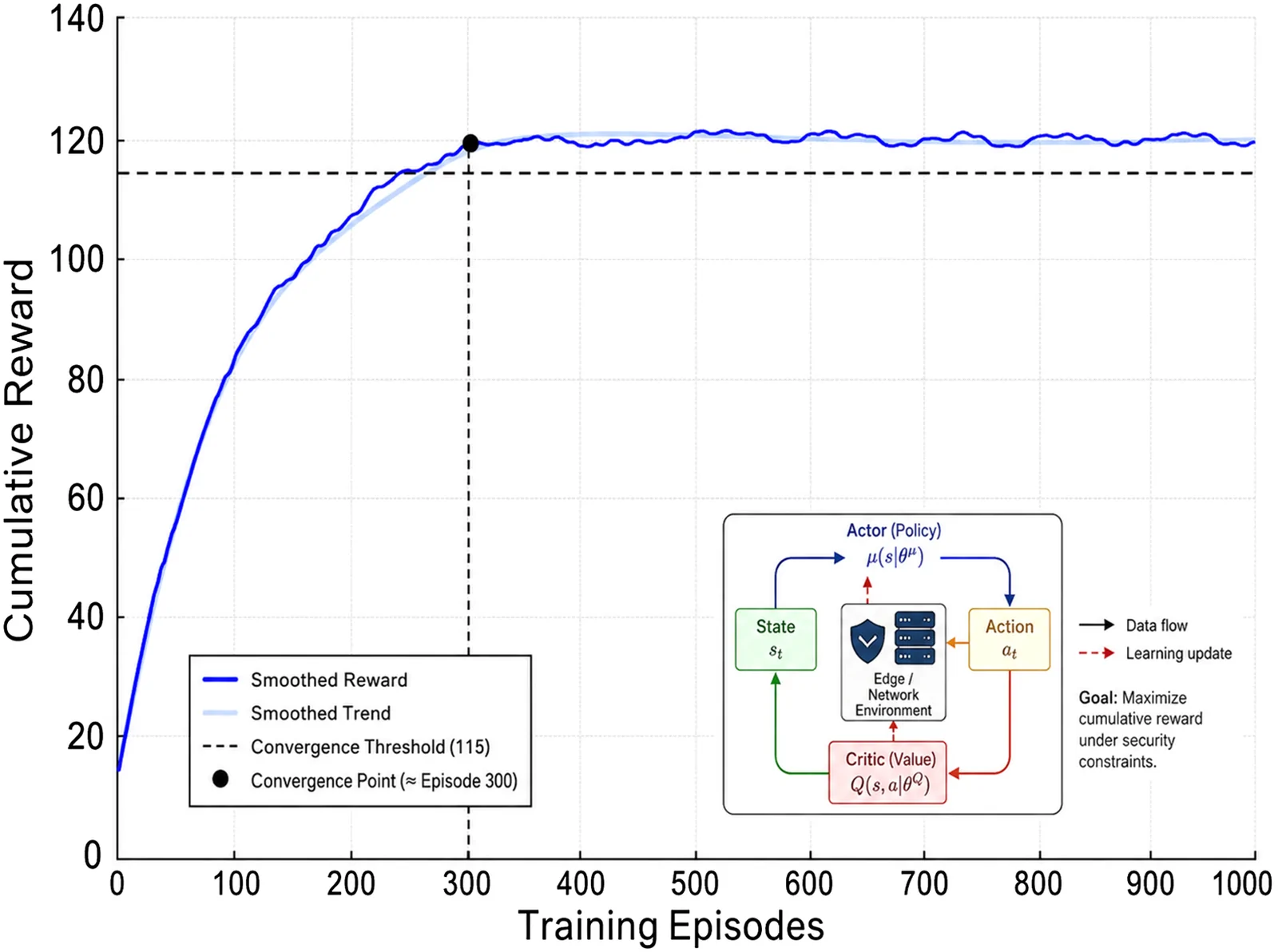
DDPG training convergence curve for the DSO-Engine The cumulative reward increases rapidly during early training and stabilizes after approximately 300 episodes, indicating convergence to a policy that balances security strength, latency, and energy overhead. State inputs include runtime resource indicators, cryptographic execution metrics, QKD key status, IDS alerts, and authentication failures; action outputs modulate encryption level, authentication mode, key-refresh mode, privacy budget, and privacy-preserving computation mode. The actor-critic architecture employs two fully connected hidden layers with ReLU activation, optimized using Adam (actor learning rate: 1e−4; critic: 1e−3; discount factor γ = 0.99; soft update τ = 0.005). All candidate actions are validated through a safe-action filter that enforces minimum cryptographic strength, authentication requirements, and privacy-budget constraints before execution.

A significant advancement is the validation of cross-platform deployability. Traditional PQC evaluations focus on high-performance servers, yet edge computing requires support for devices ranging from industrial gateways to microcontrollers. Our heterogeneous compatibility analysis shows that instruction-level optimizations and enclave-aware resource management enable efficient PQC execution across the performance spectrum. Latency remained sub-150 ms on Raspberry Pi 3B+ (158 ms) and sub-120 ms on NUC platforms, with manageable memory footprints (245 MB on Pi, 145 MB on NUC). As shown in Figure 8G, security guarantees remained consistent across platforms (scores >0.95), confirming that the architecture abstracts hardware heterogeneity to provide uniform security properties. This is crucial for industrial IoT where legacy devices coexist with modern nodes. Nevertheless, higher CPU utilization on Raspberry Pi devices (58%–85% versus 35%–55% on NUCs) suggests that severely constrained microcontrollers may require additional hardware acceleration or lighter PQC parameter sets.

The network topology adaptability analysis addresses structural resilience in distributed security. The Q-TEE architecture maintains robust performance across star, mesh, tree, ring, and hybrid configurations. Mesh topology exhibits superior scalability, with latency increasing from 88 ms to only 145 ms when scaling from 5 to 100 nodes, compared with 95 –215 ms for star topologies. As shown in Figure 9H, fault recovery times remained sub-1,000 ms for node and link failures, though QKD link failures required up to 1,200 ms due to physical-layer re-initialization. Bandwidth utilization efficiency remained high (75%–92%) across varying bandwidths, confirming that quantum-enhanced security does not impose prohibitive communication overhead even in constrained topologies. Practical QKD deployment depends on optical hardware cost, fiber distance, noise, synchronization, and detector stability. Therefore, the QKD component is best interpreted as an optional trust-enhancement layer for gateway-level links or high-value sessions, while large-scale deployment should rely on hybrid PQC-QKD operation where PQC provides baseline post-quantum key establishment and QKD supplies supplementary entropy or key-refresh support when available.

Long-term stability testing provides compelling evidence for production readiness. Over 7 days of continuous operation, the architecture maintained consistent performance (throughput ∼120 Mbps, CPU ∼65%) with minimal degradation (throughput retention >95.5% after 30 days) and high availability (99.88% ± 0.04%, 95% CI: 99.82%–99.94%). The minimal memory leak rate (2.5 MB/h) and authentication success rate (>99.6%) validate enclave implementation quality. The DSO-Engine achieved an MTBF of 850 h, and optimized PQC libraries exhibited reliability comparable to classical cryptography, addressing concerns that post-quantum algorithms might introduce instability. However, the QKD subsystem showed a lower MTBF (520 h) due to photon detector sensitivity and fiber alignment drift, suggesting that unattended deployments require periodic maintenance windows or self-calibration mechanisms. No abnormal drift was observed in policy response time, authentication throughput, or key-refresh behavior during the observation window.

Overall, coordinated optimization across quantum-enhanced trust, post-quantum cryptography, and adaptive policy control yields substantial performance and security advantages for heterogeneous, large-scale edge deployments.

## Resource availability

### Lead contact

Requests for further information and resources should be directed to and will be fulfilled by the lead contact, Yuanju Zhou (zhou_stbu@163.com).

### Materials availability

This study did not generate new unique reagents.

### Data and code availability

• Data: The data supporting the findings of this study are openly available in Figshare at https://doi.org/10.6084/m9.figshare.32627163. Data reported in this paper will be shared by the lead contact upon request.
• Code: The code supporting the findings of this study is openly available in Figshare at https://doi.org/10.6084/m9.figshare.32627163. This paper does not report original code beyond the deposited reproducibility package.
• Other items: Any additional information required to reanalyse the data reported in this paper is available from the lead contact upon request.

## Acknowledgments

This research was supported by the Chengdu Science and Technology Bureau Key R&D Support Program (Grant No. 2025-YF11-00032-HZ).

## Author contributions

Y.L. performed conceptualization, methodology, model design, experiments, writing – original draft, and revision. Y.Z. conducted data curation, formal analysis, visualization, and writing – review and editing.

## Declaration of interests

The authors declare no competing interests.

## STAR★Methods

### Key resources table

**Table:** 

REAGENT or RESOURCE	SOURCE	IDENTIFIER
**Deposited data (figshare dataset link)**		

Q-TEE-DSO reproducibility package (data and code)	This paper	Figshare: https://doi.org/10.6084/m9.figshare.32627163

**Software and algorithms**		

Ubuntu 20.04 LTS (operating system)	Canonical Ltd.	https://ubuntu.com/
Intel SGX SDK and DCAP driver	Intel Corporation	https://www.intel.com/content/www/us/en/developer/tools/software-guard-extensions/overview.html
CRYSTALS-Kyber v3.0 (post-quantum key encapsulation mechanism)	NIST/CRYSTALS Team	https://pq-crystals.org/kyber/
CRYSTALS-Dilithium v3.1 (post-quantum digital signature)	NIST/CRYSTALS Team	https://pq-crystals.org/dilithium/
BFV homomorphic encryption library (implementation used in this study)	This paper	Figshare: https://doi.org/10.6084/m9.figshare.32627163
Metasploit Framework (threat emulation)	Rapid7	https://www.metasploit.com/
Suricata (intrusion detection system)	Open Information Security Foundation	https://suricata.io/
EdgeBench benchmark suite (customized workload traces)	This paper	Figshare: https://doi.org/10.6084/m9.figshare.32627163
DSO-Engine (DDPG-based adaptive security policy optimizer)	This paper	Figshare: https://doi.org/10.6084/m9.figshare.32627163
NIST SP 800-22 randomness test suite	National Institute of Standards and Technology	https://csrc.nist.gov/projects/random-bit-generation/documentation-and-software

**Other**		

Intel NUC mini-computer (Core i7-1165G7)	Intel Corporation	https://ark.intel.com/content/www/us/en/ark/products/208921/intel-core-i7-1165g7-processor-12m-cache-up-to-4-70-ghz.html
Raspberry Pi 4 Model B	Raspberry Pi Foundation	https://www.raspberrypi.com/products/raspberry-pi-4-model-b/
FPGA-based measurement-device-independent QKD system	This paper	N/A

### Experimental model and study participant details

The experimental model was designed as a heterogeneous edge computing testbed comprising three categories of subjects: (i) 10 Intel NUC mini-computers equipped with Core i7-1165G7 processors as high-performance edge nodes; (ii) 15 Raspberry Pi 4 Model B units as resource-constrained end nodes; and (iii) an FPGA-based measurement-device-independent QKD system as the quantum key distribution source. The software environment ran Ubuntu 20.04 LTS on NUC nodes, integrating the Intel SGX SDK and DCAP driver. The Q-TEE runtime environment was developed on this platform and embedded with instruction-level optimized CRYSTALS-Kyber v3.0, Dilithium v3.1, and BFV homomorphic encryption libraries. Synthetic application workloads were generated using a customized EdgeBench benchmark suite to simulate intelligent traffic monitoring and distributed health monitoring scenarios. Threat emulation was performed using the Metasploit framework, and alert logs were captured by Suricata intrusion detection system.

### Method details

#### Quantum-enhanced trust root construction

In the zero-trust edge architecture, the initial trust root was strengthened by integrating a quantum random number generator (QRNG) into the TEE-assisted trust initialization process, rather than relying on classical pseudo-random number generators vulnerable to prediction attacks in the quantum computing era.^8^^,^^9^ The QRNG utilized the intrinsic randomness of quantum physical processes, where single-photon detection time intervals followed a Poisson distribution.^29^ By binary quantizing these intervals, high-entropy random sequences were generated.^30^^,^^31^ Von Neumann debiasing was applied to eliminate hardware bias:

(Equation 1) $$\text{VN}\left(b_{1},b_{2}\right)=b_{1},\text{if}\;b_{1}\neq b_{2};\;\text{discard},\text{if}\;b_{1}=b_{2}$$

ensuring statistical independence and uniformity of the output.^32^

The root-of-trust entropy was defined as H_TR_ = H_QRNG_ + H_phys_ + I_leak_, where H_QRNG_ is the quantum source entropy, H_phys_ is environmental entropy, and I_leak_ is potential side-channel leakage. Under ideal shielding, I_leak_→0 and H_TR_ ≈ H_QRNG_. The QRNG output seeded PQC key generation, where the Kyber key generation function KeyGen(1^λ^; r) used r←QRNG-output to ensure unpredictability.^33^ The quantum-resistant key entropy satisfied $H_{\text{key}}^{Q}\geq n-2^{-s}$ for security parameter s meeting NIST PQC Level 5 requirements. For SGX enclave sealing, the key was derived as K_seal_ = KDF(r_QRNG_, MRENCLAVE, MRSIGNER), where r_QRNG_ provided information-theoretic security.^34^ The QRNG hardware architecture is shown in Figure 11.

QRNG entropy validation was performed under non-ideal edge conditions using the NIST SP 800-22 randomness test suite, with pass rate P_pass_ = (1/N)∑I(P_i_ > 0.01). Statistical pass rates provided evidence against obvious distributional defects, but the QRNG output was further interpreted through a min-entropy perspective considering raw bit bias, short-range correlation, and environmental drift. The debiased output served as the seed source for PQC key generation, nonce initialization, and enclave sealing. The QRNG-enhanced trust root was not treated as an unconditional security guarantee; its operational security depended on the physical entropy source quality, debiasing effectiveness, and absence of direct QRNG device compromise.

#### Lightweight QKD link integration

To achieve quantum-resistant key distribution, a lightweight QKD module was integrated into the Q-TEE architecture using a simplified BB84 protocol adapted to edge network characteristics.^35^^,^^36^ QKD ensured information-theoretic security through the quantum no-cloning theorem, remaining secure against quantum computer attacks.^37^ Figure 12 illustrates the BB84-based quantum key distribution process, showing quantum bit preparation, basis selection, and measurement between transmitter (Alice) and receiver (Bob) over quantum and authenticated classical channels.

In BB84, Alice randomly selected basis b∈{Z,X} and bit m∈{0,1} to prepare quantum states |0⟩, |1⟩, |+⟩, or |−⟩. Bob randomly selected measurement basis b′, and bits with matching bases (b = b′) were retained as the raw key. Due to optical device costs and distance limitations in edge environments,^38^ QKD deployment was constrained to QKD-capable gateways, backbone links, or selected high-value edge nodes. Ordinary edge devices or QKD-unavailable links used PQC-based key encapsulation as the default fallback. This hybrid PQC-QKD design reflected practical constraints including optical hardware cost, fiber loss, synchronization, and quantum bit error rate.

A QKD-TCP/IP fusion architecture was adopted, using the QKD key as the pre-shared key for TLS 1.3.^39^^,^^40^ The effective QKD key generation rate was modeled as R_key_ = R_p_·η_det_·μ·e^-αL^·[1-2h_2_(Q)], where R_p_ is pulse rate, η_*det*_ is detection efficiency, μ is average photon number, α is fiber loss, L is distance, Q is quantum error rate, and h_2_ is binary entropy. A measurement-device-independent variant was used where intermediate node Charlie performed Bell state measurement and the key was jointly generated by Alice and Bob,^41^^,^^42^ with security constrained by I(A:B) ≥ I(A:E) + δ, where δ > 0 is the security margin.

The QKD key K_QKD_ bound TEE remote attestation by replacing the platform authentication key exchange: $K_{\text{PAK}}=\text{AES}\;-\;\text{GCM}\left(K_{\text{QKD}},\text{Nonce}\right)⊕K_{\text{PA}K_{\text{plain}}}$. Since K_QKD_ is information-theoretically secure, PAK remains confidential even if TLS is compromised. The QKD-enhanced authentication security level was defined as S_auth_ = *min*(S_QKD_, S_SGX_), where K_QKD_ = log_2_ε_*sec*_ and ε_*sec*_ is the protocol provable security bound. When QKD key generation was degraded by distance, noise, synchronization error, or hardware failure, Q-TEE-DSO switched to PQC-based fallback to maintain session continuity.

#### Post-quantum cryptographic optimization

To reduce computational overhead on edge devices, CRYSTALS-Kyber and Dilithium were optimized at the instruction level for execution within Intel SGX enclaves.^43^^,^^44^ Kyber is based on the module Learning with Errors (LWE) problem, with core operations including KeyGen, Encaps, and Decaps.^45^^,^^46^ Polynomial multiplication c = a · b mod q was accelerated using the Number Theoretic Transform (NTT):NTT(a) = Σa_i_ω^ij^ with ω as the primitive n-th root modulo q, enabling pointwise multiplication c = INTT(NTT(a)⊙NTT(b)). The NTT butterfly operation was optimized using pre-computed twiddle factor tables, reducing modular multiplications. The optimized NTT complexity was $T_{\text{NTT}}^{\text{opt}}=\left(n/2\right)\text{lo}g_{2}n\cdot \left(c_{m}+c_{a}\right)$, where c_m_ and c_a_ are modular multiplication and addition costs.

For Dilithium signatures, sparse vector pre-generation was used: a vector set V conforming to the central binomial distribution was pre-generated and indexed at runtime as y ← V[h(msg, nonce) mod |V|]. To prevent memory access pattern leakage in SGX enclaves, constant-time programming was implemented: if∗(c,x,y) = (c⋀x)⋁(¬c⋀y), eliminating branch prediction leaks.

#### TEE memory isolation and side-channel protection

Running lattice-based PQC algorithms in Intel SGX exposes cache and branch predictor side channels that may leak secret parameters through timing or cache collision analysis.^11^^,^^47^ A dual-layer protection mechanism was implemented combining constant-time programming at the logical layer with cache isolation at the physical layer. Side-channel information leakage was quantified as mutual information I(Key;Trace) = H(Key)-H(Key|Trace), with the goal of minimizing this quantity.

Cache Allocation Technology (CAT) mapped PQC private key areas to dedicated cache sets: CacheSet_i_ = addr|addrmodN = i for i ∈ [0, k−1], restricting non-enclave access. To mitigate power analysis, random noise injection was performed within the enclave: after key computation, random computation loops R executed x ← x ⊕ QRNG() for r iterations, where r ← QRNG() mod R_*max*_. This destroyed correlations between power traces and secret data.

#### Dynamic strategy optimization engine

In dynamic zero-trust edge environments, static security configurations cannot adapt to real-time threat and resource changes.^48^^,^^49^ The DSO-Engine modeled policy formulation as a Markov decision process (S, A, P, R, γ). The state space comprised s_t_ = [CPU_t_%,Mem_t_%,RTT_t_, ThreatScore_t_,Battery_t_%], where ThreatScore was calculated from five normalized indicators:

ThreatScore = 0.30 × IDS alert intensity + 0.20 × abnormal login frequency + 0.20 × remote attestation failure rate + 0.15 × network anomaly level + 0.15 × resource-pressure level.

The action space included encryption level (Kyber-512/768/1024), authentication mode, key-refresh mode, privacy budget, and privacy-preserving computation mode. The reward function balanced security, latency, and energy:

(Equation 2) $$r_{t}=\omega_{s}\cdot \sigma \left(S_{\text{level}}\left(a_{t}\right)\right)-\omega_{p}\cdot \lambda \left(\text{Latency}\left(a_{t}\right)\right)-\omega_{e}\cdot \eta \left(\text{Energy}\left(a_{t}\right)\right)$$

where ω_s_, ω_p_, and ω_e_ are weight coefficients, σ(·) is Sigmoid normalization, and λ(·) and η(·) are penalty functions.

A Deep Deterministic Policy Gradient (DDPG) agent with actor-critic architecture was trained for continuous state-action spaces.^50^^,^^51^ The actor μ(s|θˆμ) output deterministic actions, while the critic Q(s, a|θˆQ) evaluated state-action values. Target networks were updated via soft copying. The key training settings are summarized in Table 10.

The DDPG training convergence curve is shown in Figure 13. The cumulative reward increased rapidly during early training and stabilized after approximately 300 episodes, indicating that the DSO-Engine learned a stable policy for balancing security strength, latency, and energy overhead. To prevent unsafe policy exploration, all candidate actions were passed through a safe-action filter before execution. Actions violating minimum cryptographic strength, authentication requirements, or privacy-budget constraints were rejected and replaced by the nearest valid security configuration.

#### Adaptive privacy-preserving computation

To meet differentiated privacy and performance requirements, the Q-TEE architecture implemented runtime adaptive switching among three privacy computing modes under DSO-Engine control^52^^,^^53^: Local Secure Aggregation (LSA), Brakerski-Fan-Vercauteren (BFV) homomorphic encryption, and Differential Privacy Masking (DPM).

LSA was suitable for low-threat, high-efficiency scenarios, where raw data were preprocessed and aggregated within the local enclave and only aggregated results transmitted, with privacy loss ε_local_ ≈ 0. BFV homomorphic encryption supported direct computation on ciphertext for high-security scenarios. For plaintext message space Z_t_ and ciphertext c ∈ R_q_ where R_q_ = Z_q_[X]/(X^N^+1), aggregation was performed as c_sum_ = Σ c_i_ with Dec(c_sum_) = Σx_i_. DPM applied the Laplace mechanism y = y + Lap(Δf/ε) before data release, providing quantifiable (ε, δ)-privacy guarantees at the cost of some data accuracy.

The DSO-Engine selected the optimal mode m∗ = *argmax*_m_U(m|s_t_) based on utility U(m|s_t_) = α·S(m)-β·P(m|s_t_)-γC(m), where S(m) is inherent security level, P(m|s_t_) is expected performance overhead under current load, and C(m) is configuration complexity. A double-buffering mechanism (Buffer_active_↔Buffer_standby_) ensured atomic and consistent mode transitions.

#### Threat model and symbolic security analysis

The threat model assumed an adversary capable of controlling the public network, replaying or modifying messages, impersonating edge devices, compromising the operating system or hypervisor, manipulating runtime policy signals, and executing long-term quantum-capable cryptanalytic attacks. Physical extraction, hardware Trojans, compromised TEE hardware, malicious QRNG/QKD devices, and supply-chain attacks were treated as out of scope. The main attack assumptions and protection mechanisms are summarized in Table 11.

Security objectives were session-key secrecy, mutual authentication, replay resistance, and policy validity. A ProVerif-style symbolic model was used to analyze the main Q-TEE-DSO handshake, treating PQC encapsulation as a secure key-establishment primitive, signatures and attestation reports as unforgeable authentication evidence, and QRNG/QKD-derived values as fresh secret material under valid channel conditions. The verified properties are summarized in Table 12.

### Quantification and statistical analysis

System performance was quantified using the following metrics: privacy computing latency (ms), authentication energy efficiency (authentications per watt-hour), policy decision response time (ms), system throughput (Mbps), CPU utilization (%), memory consumption (MB), QKD key generation rate (bps), PQC encryption/decryption latency (ms), PQC signature/verification latency (ms), key survival rate (%) under simulated quantum attack intensities (I = 2, 4, 6, 8), key regeneration latency (ms), mean time between failures (hours), mean time to repair (minutes), and system availability (%).

Runtime data were collected at 100 ms intervals by a low-overhead C++ monitoring probe deployed within the Q-TEE enclave, recording CPU utilization, memory usage, network I/O, QKD key rate, PQC algorithm execution time, and DSO-Engine decision logs. Each experimental configuration was repeated five times under identical workload traces, device types, and threat-injection settings. Results are reported as mean ± standard deviation.

Paired two-sided t-tests were used to compare the proposed method against the strongest directly comparable baseline under the same workload trace, device type, and threat-injection setting. Statistical significance was reported at *p* < 0.05, *p* < 0.01, and *p* < 0.001. Ninety-five percent confidence intervals were computed for key baseline comparisons to strengthen statistical robustness. The evaluation covered five application scenarios, three device categories, multiple network topologies, and long-duration operation traces.

### Additional resources

No additional external resources were generated in this study.
