# Supplementary material for: Quantum-enhanced trusted execution with dynamic policy optimization for zero-trust edge computing
Source: iScience. 2026 Nov 2;29(11):117784. doi: 10.1016/j.isci.2026.117784 (PMC13756054; doi:10.1016/j.isci.2026.117784)
Supplement: Supplementary file 1 [file mmc1.pdf]

iScience, Volume *29*

Supplemental information

Quantum-enhanced trusted execution with dynamic policy optimization for zero-trust edge computing

Yi Liu and Yuanju Zhou

Quantum-enhanced trusted execution with dynamic policy optimization for zero-trust edge computing

Yi Liu and Yuanju Zhou

Document purpose

This Supplemental Information document accompanies the manuscript "Quantum-enhanced trusted execution with dynamic policy optimization for zero-trust edge computing" and provides the full details referenced in the Experimental Setup section of the main text ("The released reproducibility package provides a sanitized lite implementation for architectural validation; physical QKD hardware operations, SGX enclave calls, and low-level side-channel mitigations are represented through software abstractions or simulated interfaces (see Supplemental Information for full details)"). It describes (i) the organization of the released reproducibility package, (ii) the simulated QRNG/QKD interfaces, (iii) the SGX enclave call abstraction layer, (iv) the software representation of low-level side-channel mitigations, and (v) the training and data-collection configurations needed to re-run the lite implementation. The package, together with the data supporting the findings of this study, is openly available in Figshare at https://doi.org/10.6084/m9.figshare.32627163.

S1. Organization of the reproducibility package

The released package is a sanitized lite implementation intended for architectural validation of Q-TEE-DSO. It reproduces the control flow, interfaces, and policy-optimization logic of the full testbed implementation while replacing hardware-dependent operations (physical QKD devices, SGX enclave calls, and low-level side-channel countermeasures) with software abstractions or simulated interfaces, as summarized in Table S1. The implementation is organized into four reproducible components, matching the description in the main text:

1. **System orchestration.** Key generation, encryption/decryption, secure session management, simulated QRNG/QKD support, and PQC interfaces (CRYSTALS-Kyber v3.0, Dilithium v3.1, and BFV homomorphic encryption bindings).
2. **Workload generation.** Synthetic EdgeBench-style traces emulating intelligent traffic monitoring and distributed health monitoring scenarios, together with threat events (DoS attacks and port scans) of the kind produced by the Metasploit-based emulation on the physical testbed.
3. **DSO-Engine training.** The DDPG agent, hyperparameters, and reward logging, configured as summarized in Table S2.
4. **Security-control pipeline demonstration.** An end-to-end pipeline that consumes runtime metrics, computes the ThreatScore, applies the safe-action filter, and issues adaptive security configurations.

Table S1. Mapping between full-testbed components and their representation in the lite implementation

Full-testbed component	Representation in lite package	Validation target preserved
FPGA-based MDI-QKD system	Simulated QKD link with rate model and scripted degradation events	Key-establishment workflow; PQC fallback continuity
QRNG hardware entropy source	Simulated QRNG interface with Poisson-distributed detection intervals and Von Neumann debiasing	Trust-root initialization and PQC seeding workflow
Intel SGX enclave (ECALL/OCALL, DCAP attestation, sealing)	Enclave abstraction layer exposing identical call signatures with software-simulated returns	TCB composition, sealing/attestation control flow, cold-start sequence
Cache Allocation Technology (CAT) and noise injection	Software abstractions of cache-set partitioning and randomized noise loops	Integration of leakage control into the trusted execution workflow
C++ in-enclave runtime probe (100 ms sampling)	Trace-driven metric feed replaying the collected items of Table S3	DSO-Engine state construction and policy response timing
Suricata alert logs and threat scoring	Simulated threat-scoring engine with scripted alert intensities	ThreatScore dynamics and safe-action filtering

S2. Simulated QRNG interface

In the full testbed, the root-of-trust entropy derives from a QRNG in which single-photon detection time intervals follow a Poisson distribution; binary quantization of these intervals, followed by Von Neumann debiasing, yields the seed source for PQC key generation, nonce initialization, and enclave sealing. The lite implementation reproduces this pipeline in software: a simulated QRNG interface draws inter-arrival samples from an exponential (inter-event) distribution parameterizable by mean photon-detection rate, quantizes successive interval pairs into raw bits, and applies the same Von Neumann debiasing rule used on hardware:

$$\mathrm{VN}(b_1, b_2) = b_1 \ \text{ if } \ b_1 \neq b_2; \quad \text{discard if } \ b_1 = b_2 \tag{S1}$$

The debiased stream feeds the same three consumers as on hardware: Kyber key generation via $\mathrm{KeyGen}(1^{\lambda}; r)$ with $r \leftarrow \mathrm{QRNG{-}output}$; nonce initialization for session establishment; and the sealing key derivation $K_{seal} = \mathrm{KDF}(r_{QRNG}, \mathrm{MRENCLAVE}, \mathrm{MRSIGNER})$, which in the lite package is evaluated over simulated measurement values. Statistical quality of the simulated stream can be checked with the NIST SP 800-22 suite, using the pass-rate criterion $P_{pass} = (1/N)\,\Sigma\, I(P_i > 0.01)$ reported in the main text. Consistent with the main text, the simulated trust root is not presented as an unconditional security guarantee; the interface exists to validate the entropy-flow architecture rather than physical entropy quality.

S3. Simulated QKD link interface

The physical testbed integrates an FPGA-based measurement-device-independent (MDI) QKD system, in which an intermediate node performs Bell state measurement and the key is jointly generated by the two endpoints. The lite implementation replaces the optical layer with a simulated QKD link whose effective key-generation rate follows the same model used in the main text:

$$R_{key} = R_p \cdot \eta_{det} \cdot \mu \cdot e^{-\alpha L} \cdot [1 - 2h_2(Q)] \tag{S2}$$

where R_p is the pulse rate, η_{det} the detection efficiency, μ the average photon number, α the fiber loss coefficient, L the link distance, Q the quantum bit error rate, and h_2 the binary entropy function. All parameters are configurable, and scripted degradation events (distance increase, noise injection, synchronization error, or simulated hardware failure) drive R_{key} below the usable threshold, triggering the same PQC-based fallback path implemented on the testbed to maintain session continuity. Delivered keys are represented as 256-bit simulated QKD keys and are bound to the (abstracted) remote-attestation flow exactly as in the full system: the QKD key serves as the pre-shared key for TLS 1.3 in the QKD-TCP/IP fusion architecture, and the platform authentication key is wrapped as $K_{PAK} = \mathrm{AES{-}GCM}(K_{QKD}, \mathrm{Nonce}) \oplus K_{PAK\_plain}$. This allows architectural validation of the hybrid PQC-QKD operation described in the Discussion, in which PQC provides baseline post-quantum key

establishment and QKD supplies supplementary entropy or key-refresh support when available.

S4. SGX enclave call abstraction

On the NUC edge nodes, the Q-TEE runtime executes inside Intel SGX enclaves using the Intel SGX SDK and DCAP driver. The enclave-side trusted computing base (TCB) comprises only the Q-TEE trusted core, the sealing/attestation interface, the optimized Kyber/Dilithium routines, the privacy-computing selector, and the DSO inference interface (TCB size 9.3 MB; peak enclave memory usage 58.7 ± 4.6 MB on NUC nodes; Raspberry Pi client-side trusted runtime footprint 36.2 ± 3.1 MB). The lite package abstracts this boundary behind an enclave interface layer that preserves the call signatures and control flow of the real ECALLs/OCALLs while returning software-simulated results. The abstraction covers four groups of calls:

- **Lifecycle calls.** Enclave creation, trusted-stack loading, and teardown, reproducing the cold-start sequence (enclave initialization, PQC library loading, sealing-key recovery, and DSO policy-state loading) whose latency on hardware averaged 214.8 ± 18.5 ms on NUC nodes and 392.6 ± 34.7 ms on Raspberry Pi terminals.
- **Attestation calls.** Quote generation and verification shims that emulate the DCAP attestation exchange and accept the (simulated) QKD-bound platform authentication key, so that the mutual-authentication and session-binding workflow can be exercised end to end.
- **Sealing calls.** Seal/unseal operations keyed by the simulated derivation $K_{seal} = KDF(r_{QRNG}, MRENCLAVE, MRSIGNER)$, preserving the sealed key-handling behavior against privileged-software compromise in the threat model.
- **Cryptographic and policy calls.** Entry points for the instruction-level optimized Kyber/Dilithium operations and for DSO policy inference, so that policy decisions traverse the same enclave boundary as on hardware.

The abstraction layer is deliberately signature-compatible: the same orchestration code paths run against the abstraction in the lite package and against the SGX SDK on the testbed, which is what permits architectural validation without SGX-capable hardware.

S5. Software representation of side-channel mitigations

The full implementation combines constant-time programming at the logical layer with cache isolation at the physical layer, quantifying residual leakage as the mutual information $I(Key; Trace) = H(Key) - H(Key \mid Trace)$. In the lite package these low-level mitigations are represented through software abstractions:

- **Constant-time selection.** Branch-free conditional selection $\text{if}*(c, x, y) = (c \wedge x) \oplus (\neg c \wedge y)$ is retained verbatim in the PQC code paths, since it is portable C and eliminates branch-prediction leakage independently of the platform.
- **Cache isolation abstraction.** Cache Allocation Technology (CAT) partitioning of PQC private-key regions into dedicated cache sets ($CacheSet_i = \{addr \mid addr \bmod N = i\}$ for $i \in [0, k-1]$) is represented by a logical partitioning interface with the same configuration surface; on non-CAT hardware the calls are accepted and logged so that the isolation workflow is exercised without enforcing physical set partitioning.
- **Noise-injection abstraction.** The randomized noise loop executed after key computation ($x \leftarrow x \oplus QRNG()$ for r iterations, with $r \leftarrow QRNG() \bmod R_{max}$) is preserved using the simulated QRNG source of Section S2, maintaining the power-analysis countermeasure's control flow and its consumption of quantum randomness.

Scope note. *Consistent with the main text (Table 3), side-channel resistance is not modeled symbolically and is evaluated separately on the physical testbed; the lite abstractions validate the integration of leakage control into the trusted execution workflow, not the physical leakage bounds themselves.*

S6. DSO-Engine training configuration

The DSO-Engine formulates policy optimization as a Markov decision process (S, A, P, R, γ) and is trained with a Deep Deterministic Policy Gradient (DDPG) agent using an actor-critic architecture with soft-updated target networks. The state vector is s_t = [CPU_t%, Mem_t%, RTT_t, $ThreatScore_t$, $Battery_t$%], where the ThreatScore is computed from five normalized indicators (0.30 × IDS alert intensity + 0.20 × abnormal login frequency + 0.20 × remote attestation failure rate + 0.15 × network anomaly level + 0.15 × resource-pressure level). The reward balances security, latency, and energy:

$$r_t = \omega_s \cdot \sigma(S_{level}(a_t)) - \omega_p \cdot \lambda(Latency(a_t)) - \omega_e \cdot \eta(Energy(a_t)) \quad (S3)$$

with Sigmoid normalization $\sigma(\cdot)$ and penalty functions $\lambda(\cdot)$, $\eta(\cdot)$. All candidate actions pass through the safe-action filter before execution; actions violating minimum cryptographic strength, authentication requirements, or privacy-budget constraints are rejected and replaced. The key training settings, identical to Table 1 of the main text, are reproduced in Table S2 for completeness, since the training component of the package loads exactly this configuration.

Table S2. Key reproducibility settings of the DSO-Engine (as Table 1 of the main text)

Item	Configuration
State inputs	CPU utilization, memory usage, network throughput, QKD key rate, PQC latency, IDS alerts, authentication failures, and policy-switching history
Action outputs	Encryption level, authentication mode, key-refresh mode, privacy budget, and privacy-preserving computation mode
Actor network	Two fully connected hidden layers with ReLU activation
Critic network	State-action network with two fully connected hidden layers and ReLU activation
Optimizer	Adam
Learning rates	Actor: 1e-4; Critic: 1e-3
Discount factor and soft update	$\gamma = 0.99$; $\tau = 0.005$
Training batch and memory	Mini-batch size: 64; replay memory: 100,000 transitions
Exploration strategy	Action noise with standard deviation of 0.1
Security configuration range	Kyber-512/768/1024, Dilithium levels 2/3/5, 256-bit simulated QKD key, and privacy-budget adjustment

S7. Runtime metrics consumed by the lite implementation

The security-control pipeline demonstration replays the runtime metrics collected on the testbed by the low-overhead C++ probe deployed inside the Q-TEE enclave. Table S3 (Table 4 of the main text) lists the collected items and sampling rates; the same schema defines the trace format accepted by the lite package, so that testbed-recorded traces and synthetic EdgeBench-style traces are interchangeable.

Table S3. Collected runtime metrics and sampling rates (as Table 4 of the main text)

Collected metric	Definition and description	Sampling rate
CPU utilization (%)	Average edge node processor utilization, reflecting computational load	100 ms
Memory usage (MB)	Physical memory occupied by Q-TEE core components (enclaves, DSO-Engine, and PQC libraries)	100 ms
Network I/O (KB/s)	Node network input/output bandwidth, used to measure	100 ms

Collected metric	Definition and description	Sampling rate
	communication overhead	
QKD key generation rate (bps)	Bit rate of the shared key successfully distributed by the QKD link for encryption	1 s
PQC encryption and decryption latency (ms)	Time required for a complete Kyber key encapsulation or decapsulation operation	Event-triggered (per operation)
PQC signature verification latency (ms)	Time required for a complete Dilithium signature generation and verification operation	Event-triggered (per operation)

S8. Relationship to the full testbed and limitations

The lite implementation is intended for architectural validation: it reproduces the cross-layer orchestration among quantum-derived trust establishment, TEE-protected execution, optimized PQC operation, and DSO-Engine policy control, and it allows third parties to re-run policy training and the security-control pipeline without access to QKD optics or SGX-capable hardware. It does not reproduce hardware-dependent measurements — physical QKD key rates and quantum bit error rates, enclave memory footprints, cold-start latencies, side-channel leakage levels, and long-duration reliability figures (e.g., component MTBF) reported in the main text were all measured on the physical testbed described in the STAR Methods and are not regenerated by the lite package. Data underlying those measurements are available in the same Figshare deposit (https://doi.org/10.6084/m9.figshare.32627163). Any additional information required to reanalyse the data reported in this paper is available from the lead contact upon request.
